# Identification of Conserved Moieties in Metabolic Networks by Graph Theoretical Analysis of Atom Transition Networks

**DOI:** 10.1371/journal.pcbi.1004999

**Published:** 2016-11-21

**Authors:** Hulda S. Haraldsdóttir, Ronan M. T. Fleming

**Affiliations:** Luxembourg Centre for Systems Biomedicine, University of Luxembourg, Esch-sur-Alzette, Luxembourg; The Pennsylvania State University, UNITED STATES

## Abstract

Conserved moieties are groups of atoms that remain intact in all reactions of a metabolic network. Identification of conserved moieties gives insight into the structure and function of metabolic networks and facilitates metabolic modelling. All moiety conservation relations can be represented as nonnegative integer vectors in the left null space of the stoichiometric matrix corresponding to a biochemical network. Algorithms exist to compute such vectors based only on reaction stoichiometry but their computational complexity has limited their application to relatively small metabolic networks. Moreover, the vectors returned by existing algorithms do not, in general, represent conservation of a specific moiety with a defined atomic structure. Here, we show that identification of conserved moieties requires data on reaction atom mappings in addition to stoichiometry. We present a novel method to identify conserved moieties in metabolic networks by graph theoretical analysis of their underlying atom transition networks. Our method returns the exact group of atoms belonging to each conserved moiety as well as the corresponding vector in the left null space of the stoichiometric matrix. It can be implemented as a pipeline of polynomial time algorithms. Our implementation completes in under five minutes on a metabolic network with more than 4,000 mass balanced reactions. The scalability of the method enables extension of existing applications for moiety conservation relations to genome-scale metabolic networks. We also give examples of new applications made possible by elucidating the atomic structure of conserved moieties.

## Author Summary

Conserved moieties are transferred between metabolites in internal reactions of a metabolic network but are not synthesised, degraded or exchanged with the environment. The total amount of a conserved moiety in the metabolic network is therefore constant over time. Metabolites that share a conserved moiety have interdependent concentrations because their total amount is constant. Identification of conserved moieties results in a concise description of all concentration dependencies in a metabolic network. The problem of identifying conserved moieties has previously been formulated in terms of the stoichiometry of metabolic reactions. Methods based on this formulation are computationally intractable for large networks. We show that reaction stoichiometry alone gives insufficient information to identify conserved moieties. By first incorporating additional data on the fate of atoms in metabolic reactions, we developed and implemented a computationally tractable algorithm to identify conserved moieties and their atomic structure.

This is a *PLOS Computational Biology* Methods paper.

## Introduction

Conserved moieties give rise to pools of metabolites with constant total concentration and dependent individual concentrations. These constant metabolite pools often consist of highly connected cofactors that are distributed throughout a metabolic network. Representative examples from energy metabolism include the AMP and NAD moieties [1, 2]. Changes in concentration ratios within these cofactor pools affect thermodynamic and mass action kinetic driving forces for all reactions they participate in. Moiety conservation therefore imposes a purely physicochemical form of regulation on metabolism that is mediated through changes in concentration ratios within constant metabolite pools. Reich and Sel’kov likened conserved moieties to turning wheels that are “geared into a clockwork” [2]. They described the thermodynamic state of energy metabolism as “open flow through a system closed by moiety conservation”. Identification of conserved moieties in metabolic networks has helped elucidate complex metabolic phenomena including synchronisation of glycolytic oscillations in yeast cell populations [3] and the function of glycosomes in the African sleeping sickness parasite *Trypanosoma brucei* [4]. It has also been shown to be relevant for drug development [4, 5].

Identification of conserved moieties has been of interest to the metabolic modelling community for several decades [6, 7]. It is particularly important for dynamic modelling [8] and metabolic control analysis [9] where metabolite concentrations are explicitly modelled. Moiety conservation relations provide a sparse, physically meaningful description of concentration dependencies in a metabolic network. They can be used to eliminate redundant metabolite concentrations as the latter can be derived from the set of independently varying metabolite concentrations. Doing so facilitates simulation of metabolic networks and is in fact required for many computational modelling methods [6, 7].

**Fig 1:**
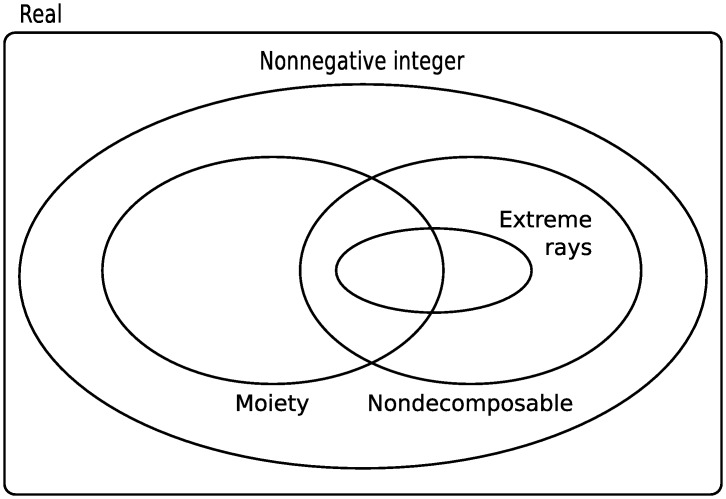
Sets of conservation vectors for metabolic networks. The set of real-valued conservation vectors consists of all vectors in the left null space of a stoichiometric matrix. Real-valued basis vectors can be computed using efficient linear algebra algorithms but are difficult to interpret as they generally contain negative and noninteger elements. Nonnegative integer vectors are easier to interpret but more difficult to compute. Existing algorithms have exponential worst case time complexity. Algorithms exist to compute extreme rays, the set of all nondecomposable nonnegative integer vectors, and a maximal set of linearly independent nonnegative integer vectors. These vector sets intersect with the set of moiety vectors but are not equivalent to it. Moiety vectors represent conservation of an identifiable group of atoms in network metabolites. They are a property of the specific set of metabolites and reactions that constitute a metabolic network whereas other conservation vectors are a property of the network’s stoichiometric matrix. The method presented here computes moiety vectors in polynomial time.

Mathematically, moiety conservation gives rise to a stoichiometric matrix with linearly dependent rows. The left null space of the stoichiometric matrix therefore has nonzero dimension (see Theoretical Framework, Section Moiety vectors). Vectors in the left null space, hereafter referred to as conservation vectors, can be divided into several interrelated sets based on their numerical properties and biochemical meaning (Fig 1). *Moiety vectors* constitute a subset of conservation vectors with a distinct biochemical interpretation. Each moiety vector represents conservation of a particular metabolic moiety. Elements of a moiety vector correspond to the number of instances of a conserved moiety in metabolites of a metabolic network. As moieties are discrete quantities, moiety vectors are necessarily nonnegative integer vectors.

Methods exist to compute conservation vectors based only on the stoichiometric matrix of a metabolic network. These methods compute different types of bases for the left null space of the stoichiometric matrix (see S1 Appendix for mathematical definitions). Each method draws basis vectors from a particular set of conservation vectors (Fig 1). There is a tradeoff between the computational complexity of these methods and the biochemical interpretability of the basis vectors they return. At the low end of the computational complexity spectrum are linear algebraic methods such as singular value decomposition. Other methods, such as Householder QR factorisation [7] or sparse LU factorisation [10] are more efficient for large stoichiometric matrices. These methods construct a linear basis for the left null space from real-valued conservation vectors. Though readily computed, these vectors are also the most difficult to interpret as they generally contain negative and noninteger elements.

Schuster and Höfer [11] introduced the use of vertex enumeration algorithms to compute the *extreme rays* of the positive orthant of the left null space. They referred to these extreme rays as “extreme semipositive conservation relations”. Famili and Palsson [12] later referred to them as “metabolic pools” and the set of all extreme rays as “a *convex basis* for the left null space”. Like moiety vectors, extreme rays are nonnegative integer vectors. They are therefore readily interpreted in terms of constant metabolite pools. However, extreme rays can currently only be computed for relatively small metabolic networks due to the computational complexity of vertex enumeration algorithms [13]. Moreover, the set of extreme rays is not identical to the set of moiety vectors (Fig 1). Schuster and Hilgetag [14] presented examples of extreme rays that did not represent moiety conservation relations, as well as moiety vectors that were not extreme rays.

Moiety vectors are a property of a metabolic network while extreme rays are a property of its stoichiometric matrix. Multiple metabolic networks could in theory have the same stoichiometric matrix, despite consisting of different sets of metabolites and reactions. These networks would all have the same set of extreme rays, but could have different sets of moiety vectors. Schuster and Hilgetag [14] published an extension to the vertex enumeration algorithm in [11] to compute the set of all *nondecomposable nonnegative integer vectors* in the left null space of a stoichiometric matrix. This set is guaranteed to contain all nondecomposable moiety vectors for a particular metabolic network as subset (Fig 1). However, it is impossible to identify the subset of moiety vectors without information about the atomic structure of metabolites.

Alternatives to vertex enumeration have been proposed to speed up computation of biochemically meaningful conservation vectors, e.g., [15–17]. Most recently, De Martino et al. [17] published a novel method to compute a *nonnegative integer basis* for the left null space of a stoichiometric matrix. This method [17] relies on stochastic algorithms, without guaranteed convergence, but that were empirically shown to perform well even on large networks. Like extreme rays, the nonnegative integer vectors computed with this method are not necessarily moiety vectors (Fig 1). In general, methods to analyse stoichiometric matrices are not suited to specifically compute moiety vectors. Computation of moiety vectors requires information about the atomic composition of metabolites. To our knowledge, only one method has previously been published to specifically compute moiety vectors for metabolic networks [18]. This method was based on nonnegative integer factorisation of the elemental matrix; a numerical representation of metabolite formulas. Nonnegative integer factorisation of a matrix is at least NP-hard [19] and no polynomial time algorithm is known to exist for this problem. Moreover, only the chemical formula but not the atomic identities of the conserved moieties can be derived from this approach. Identifying the atoms that belong to each moiety requires additional information about the fate of atoms in metabolic reactions. This information is not contained in a stoichiometric matrix.

Here, we propose a novel method to identify conserved moieties in metabolic networks. Our method is based on the premise that atoms within the same conserved moiety follow identical paths through a metabolic network. Given data on which substrate atoms map to which product atoms in each metabolic reaction, the paths of individual atoms through a metabolic network can be encoded in an *atom transition network*. Until recently, the necessary data were difficult to obtain but relatively efficient algorithms have now become available to predict atom mappings in metabolic reactions [20–22]. These algorithms have made it possible to construct atom transition networks for large metabolic networks. Unlike metabolic networks, atom transition networks are amenable to analysis with efficient graph theory algorithms. Here, we take advantage of this fact to identify conserved moieties in metabolic networks in polynomial time. Furthermore, starting from atom transition networks allows us to associate each conserved moiety with a specific group of atoms in a subset of metabolites in a metabolic network.

This work combines elements of biochemistry, linear algebra and graph theory. We have made an effort to accommodate readers from all fields. The main text consists of informal descriptions of our methods and results, accompanied by illustrative examples and a limited number of mathematical equations. Formal definitions of italicised terms are given in supporting file S1 Appendix. We precede our results with a section on the theoretical framework for this work, where we introduce key concepts and notation used in the remainder of the text.

## Theoretical Framework

### Metabolic networks

**Fig 2:**
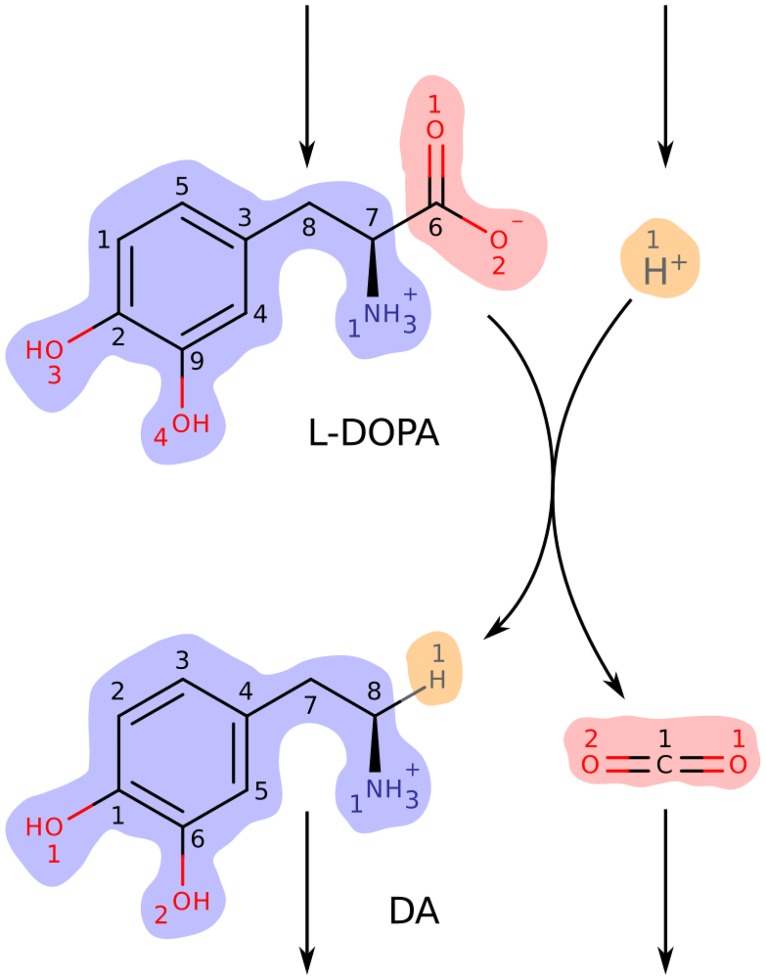
A metabolic map for an example metabolic network. The network consists of one internal reaction and four exchange reactions. The internal reaction is the DOPA decarboxylase reaction (VMH [23] ID: 3HLYTCL) that produces dopamine (DA, VMH ID: dopa) and CO_2_ (VMH ID: co2) from levodopa (L-DOPA, VMH ID: 34dhphe) and H^+^ (VMH ID: h). The open network includes source reactions for the two substrates and sink reactions for the two products. Arrowheads indicate reaction directionality. Metabolite structures were rendered from molfiles (Accelrys, San Diego, CA) with MarvinView (ChemAxon, Budapest, Hungary). Atoms are numbered according to their order in each metabolite’s molfile. Atoms of different elements are numbered separately, in colours matching their elemental symbol. The internal reaction conserves three metabolic moieties. Atoms belonging to the same moiety have identically coloured backgrounds. Levodopa and dopamine each contain one instance of a dopamine moiety (blue background). Implicit hydrogen atoms on both metabolites are also part of this moiety. Levodopa and CO_2_ each contain one instance of a CO_2_ moiety (red background). Finally, the hydrogen ion and dopamine each contain one instance of a hydrogen moiety (orange background).

A metabolic network consists of a set of metabolites that interconvert via a set of metabolic reactions. Metabolic networks in living beings are open systems that exchange mass and energy with their environment. For modelling purposes, the boundary between system and environment can be defined by introducing a set of metabolite sources and sinks collectively known as exchange reactions. Unlike internal reactions, exchange reactions are artificial constructs that do not conserve mass or charge. The topology of a metabolic network can be represented in several ways. Here, we use metabolic maps and stoichiometric matrices. A metabolic map for a small example metabolic network is shown in Fig 2. This example will be used throughout this section to demonstrate key concepts relevant to this work.

A stoichiometric matrix for an open metabolic network with *m* metabolites and *n* reactions is denoted by $S\in R^{m\times n}$. Each row of *S* represents a metabolite and each column a reaction such that element *S*_*ij*_ is the stoichiometric coefficient of metabolite *i* in reaction *j*. Coefficients are negative for substrates and positive for products. Substrates and products in reversible reactions are defined by designating one direction as forward. The stoichiometric matrix can be written as

$$S=\left[N,B\right],$$ (1)

where $N\in Z^{m\times u}$ consists of columns representing internal (mass balanced) reactions and $B\in R^{m\times (n-u)}$ consists of columns representing exchange reactions (mass imbalanced). Note that *N* represents a metabolic network that is closed to the environment. In what follows we will refer to *N* as the internal stoichiometric matrix, *B* as the exchange stoichiometric matrix, and *S* as the total stoichiometric matrix. The total stoichiometric matrix for the example metabolic network in Fig 2 is given in Table 1.

**Table 1:** The total stoichiometric matrix *S* = [*N*, *B*] for the example metabolic network.

	*N*	*B*			
L-DOPA	-1	1	0	0	0
H^+^	-1	0	1	0	0
DA	1	0	0	-1	0
CO_2_	1	0	0	0	-1

Rows are labelled with the corresponding metabolite identifier from Fig 2. The internal stoichiometric matrix $N\in Z^{4\times 1}$ is row rank deficient, with rank (*N*) = 1. The dimension of its left null space is therefore $\text{dim }(N(N^{T}))=4-1=3$. The total stoichiometric matrix $S\in Z^{4\times 5}$ is full row rank. Its left null space is therefore zero dimensional.

Stoichiometric matrices are *incidence matrices* for generalised *graphs* known as *hypergraphs* [24]. Hypergraphs contain hyperedges that can connect more than two nodes. The metabolic map in Fig 2 is a planar visualisation of a hypergraph with one hyperedge, connecting four metabolites. A graph edge that only connects two nodes is a special instance of a hyperedge. Apart from the occasional isomerisation reaction, metabolic reactions involve more than two metabolites. As a result, they cannot be represented as graph edges without loss of information. Metabolic networks are therefore represented as hypergraphs where nodes represent metabolites and hyperedges represent reactions. Since reactions have a designated forward direction, they are *directed hypergraphs*. Representing metabolic networks as hypergraphs has the advantage of conserving basic structure and functional relationships. The disadvantage is that many graph theoretical algorithms are not applicable to hypergraphs [24].

### Moiety vectors

An internal stoichiometric matrix $N\in Z^{m\times u}$ for a closed metabolic network is always row-rank deficient, i.e., rank(*N*) < *m* [11]. The left null space of *N*, denoted by $N(N^{T})$, therefore has finite dimension given by $\text{dim}(N(N^{T}))=m-\text{rank}(N)$. The left null space holds all conservation vectors for a stoichiometric matrix [8]. The number of linearly independent conservation vectors for a closed metabolic network is $\text{dim}(N(N^{T}))$. The total stoichiometric matrix *S* for an open metabolic network has a greater rank than the internal stoichiometric matrix *N* for the corresponding closed metabolic network (e.g., Table 1), i.e., rank(*N*) < rank(*S*). Consequently, $\text{dim}(N(S^{T}))<\text{dim}(N(N^{T}))$, meaning that there are fewer linearly independent conservation vectors for an open metabolic network than the corresponding closed network. This is consistent with physical reality, since mass can flow into and out of open networks but is conserved within closed networks. All quantities that are conserved in an open metabolic network are also conserved in the corresponding closed network. That is, if *z* is a conservation vector for an open metabolic network *S*, such that *S*^*T*^ *z* = 0, then *z* is also a conservation vector for the corresponding closed network *N*, and *N*^*T*^ *z* = 0, since *S* = [*N*, *B*]. The set of conservation relations for an open network is therefore a subset of all conservation relations for the corresponding closed network, i.e., $N(S^{T})\subseteq N(N^{T})$. In what follows we will mainly be concerned with the larger set of conservation relations for a closed metabolic network.

Schuster and Hilgetag [14] defined a moiety vector *l*_1_ as a nonnegative integer vector in the left null space of a stoichiometric matrix, i.e.,

$$N^{T}l_{1}=0,$$ (2)

$$l_{1}\in N_{0}^{m}.$$ (3)

In addition, they defined *l*_1_ to be a maximal moiety vector if it cannot be decomposed into two other vectors *l*_2_ and *l*_3_ that satisfy Eqs 2 and 3, i.e., if

$$l_{1}\neq \alpha_{2}l_{2}+\alpha_{3}l_{3},$$ (4)

where $\alpha_{2},\alpha_{3}\in N_{+}$. We propose a more specific definition. The properties above define increasingly small sets of conservation vectors (Fig 1). Eq 2 defines the set of all conservation vectors. Addition of Eq 3 defines the set of nonnegative integer conservation vectors and addition of Eq 4 defines the set of nonnegative integer conservation vectors that are nondecomposable. Although this set includes all nondecomposable moiety vectors as subset it is not equivalent (Fig 1). To define the set of moiety vectors we require a fourth property. We define *l*_1_ to be a moiety vector if it satisfies Eqs 2 and 3 and represents conservation of a specific metabolic moiety, i.e., an identifiable group of atoms in network metabolites. Element *l*_1,*i*_ should correspond to the number of instances of the conserved moiety in metabolite *i*. We define *l*_1_ to be a *nondecomposable moiety vector* if it satisfies condition 4 and a *composite moiety vector* if it does not. Nondecomposable moiety vectors for the DOPA decarboxylase reaction from the example metabolic network in Fig 2 are given in Table 2a. For comparison, conservation vectors computed with existing methods for conservation analysis of metabolic networks are given in Table 2b–2d. In general, these vectors do not represent moiety conservation.

**Table 2:** Different types of conservation vectors for the DOPA decarboxylase reaction.

	(a)			(b)				(c)			(d)			
	*l*_1_	*l*_2_	*l*_3_	C	H	O	N	*z*_1_	*z*_2_	*z*_3_	*l*_1_	*l*_2_	*l*_3_	*z*_4_
L-DOPA	1	1	0	9	11	4	1	-1/2	1/2	1/2	1	1	0	0
H^+^	0	0	1	0	1	0	0	5/6	1/6	1/6	0	0	1	1
DA	1	0	1	8	12	2	1	1/6	5/6	-1/6	1	0	1	0
CO_2_	0	1	0	1	0	2	0	1/6	-1/6	5/6	0	1	0	1

Moiety vectors are denoted *l*_*k*_. (a) Moiety vectors computed with the method presented here. Each column represents the conservation of a particular metabolic moiety. *l*_1_ represents conservation of the dopamine moiety (blue background in Fig 2), *l*_2_ the CO_2_ moiety (red background), and *l*_3_ the hydrogen moiety (orange background). (b) The elemental matrix. Each column represents conservation of a particular element. Elemental conservation vectors generally do not span the left null space of a stoichiometric matrix. (c) Real-valued conservation vectors computed with singular value decomposition of the internal stoichiometric matrix *N* in Table 1. Real-valued conservation vectors cannot generally be interpreted in terms of conserved moieties as they contain negative and noninteger values. (d) Extreme rays of the left null space $N(N^{T})$. The first three belong to the intersection between the sets of extreme rays and moiety vectors in Fig 1. The fourth belongs to the set difference. It cannot represent moiety conservation as no atoms are exchanged between H^+^ and CO_2_. Without information about atom mappings between metabolites it would be impossible to determine which extreme rays correspond to conserved moieties. The full set of all nondecomposable nonnegative integer vectors includes 13 additional vectors (not shown), none of which represent moiety conservation.

### Atom transition networks

**Fig 3:**
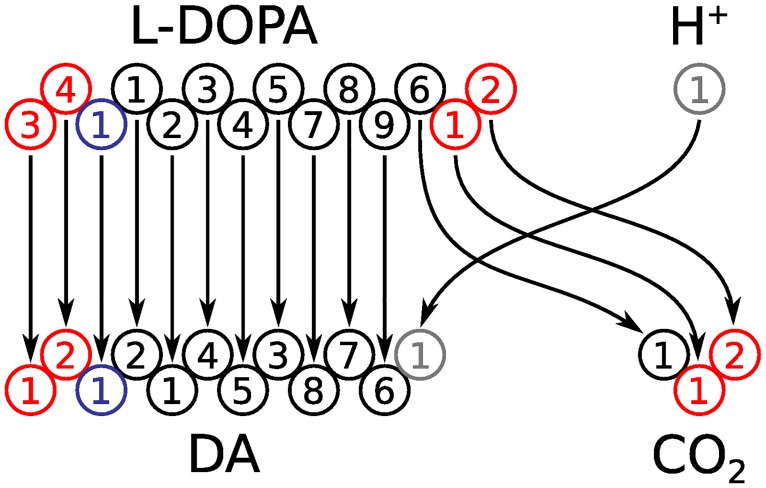
A graphical representation of an atom transition network for the DOPA decarboxylase reaction. Nodes (open circles) represent atoms. Atoms can be matched to metabolite structures in Fig 2 on their metabolite identifiers, colours and numbers. Directed edges (arrows) represent atom transitions. All except one hydrogen atom are omitted to simplify the figure.

Metabolic reactions conserve mass and chemical elements. Therefore, there must exist a mapping from each atom in a reactant metabolite to a single atom of the same element in a product metabolite. An atom transition is a single mapping from a substrate to a product atom. An *atom transition network* contains information about all atom transitions in a metabolic network. It is a mathematical structure that enables one to trace the paths of each individual atom through a metabolic network. An atom transition network can be generated automatically from a stoichiometric matrix for a metabolic network and atom mappings for internal reactions. The atom transition network for the DOPA decarboxylase reaction from the example metabolic network in Fig 2 is shown in Fig 3. Unlike metabolic networks, atom transition networks are graphs since every atom transition (edge) connects exactly two atoms (nodes). They are *directed graphs* since every atom transition has a designated direction that is determined by the directionality of the parent metabolic reaction, i.e., the designation of substrates and products. Because atom transition networks are graphs, they are amenable to analysis with efficient graph algorithms that are not generally applicable to metabolic networks due to the presence of hyperedges [24].

## Results

### Identification of conserved moieties in the dopamine synthesis pathway

**Fig 4:**
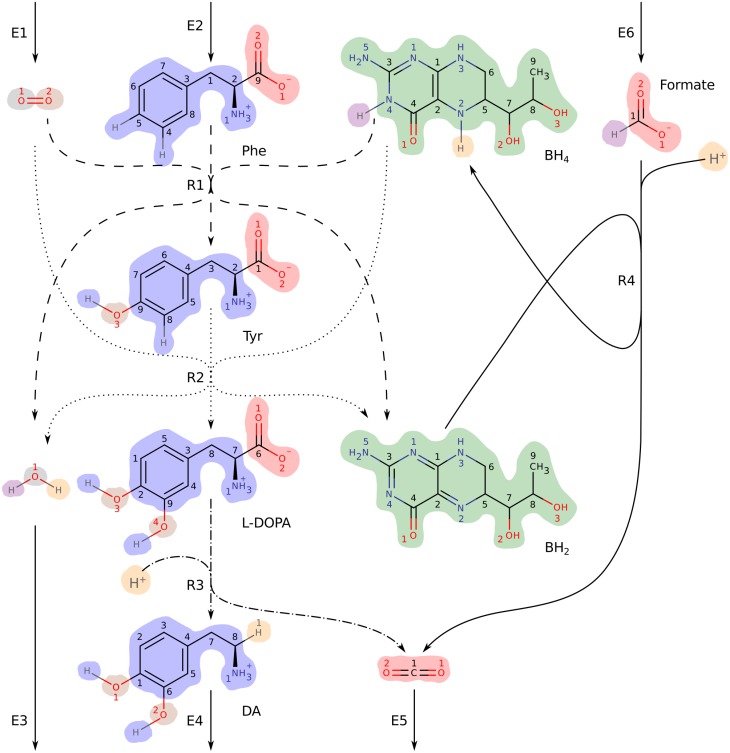
DAS: a small metabolic network consisting of reactions in the human dopamine synthesis pathway. Metabolite abbreviations are, Phe: L-phenylalanine (VMH [23] ID: phe_L), Tyr: L-tyrosine (VMH ID: tyr_L), L-DOPA: levodopa (VMH ID: 34dhphe), DA: dopamine (VMH ID: dopa), BH_4_: tetrahydrobiopterin (VMH ID: thbpt), BH_2_: dihydrobiopterin (VMH ID: dhbpt). Internal reactions are labelled R1–R4. R1 (dashed lines) is the phenylalanine hydroxylase reaction (VMH ID: r0399). R2 (dotted lines) is the tyrosine hydroxylase reaction (VMH ID: TYR3MO2 and THBPT4ACAMDASE). R3 (dash-dotted lines) is the DOPA decarboxylase reaction (VMH ID: 3HLYTCL). R4 (solid line) is a composite of the formate dehydrogenase reaction (VMH ID: FDH) and the dihydropteridine reductase reaction (VMH ID: DHPR). Exchange reactions are labelled E1–E6. The hydrogen ion (H^+^) exchange reaction E7 was omitted to simplify the figure. Atoms are numbered according to their order in each metabolite’s molfile. Atoms of different elements are numbered separately, in colours matching their elemental symbol. Atoms belonging to the same conserved moiety have identically coloured backgrounds.

We will demonstrate our method by identifying conserved moieties in the simple dopamine synthesis network DAS in Fig 4. This network consists of 11 metabolites, four internal reactions and seven exchange reactions. The total stoichiometric matrix *S* = [*N*, *B*] is given in Table 3. The internal stoichiometric matrix *N* is row rank deficient with rank (*N*) = 4. The dimension of the left null space is therefore $\text{dim}(N(N^{T}))=7$, meaning that there are seven linearly independent conservation vectors for the closed metabolic network. Our analysis of an atom transition network for DAS will conclude with the computation of seven linearly independent moiety vectors that span $N(N^{T})$. To compute these vectors we require the internal stoichiometric matrix in Table 3 and atom mappings for the four internal reactions. Here, we used algorithmically predicted atom mappings [20]. These data are required to generate an atom transition network for DAS (see Methods, Section Generation of atom transition networks). By graph theoretical analysis of this atom transition network we derive the first of two alternative representations of moiety conservation relations which we term *moiety graphs*. Nodes in a moiety graph represent separate instances of a conserved moiety. Each node is associated with a specific set of atoms in a particular metabolite. The second representation of moiety conservation relations are the moiety vectors which can be derived from moiety graphs in a straightforward manner. Moiety vectors computed with our method are therefore associated with specific atoms via moiety graphs.

**Table 3:** The total stoichiometric matrix *S* = [*N*, *B*] for DAS.

	R1	R2	R3	R4	E1	E2	E3	E4	E5	E6	E7
Phe	-1	0	0	0	0	1	0	0	0	0	0
Tyr	1	-1	0	0	0	0	0	0	0	0	0
L-DOPA	0	1	-1	0	0	0	0	0	0	0	0
DA	0	0	1	0	0	0	0	-1	0	0	0
CO_2_	0	0	1	1	0	0	0	0	-1	0	0
Formate	0	0	0	-1	0	0	0	0	0	1	0
BH_4_	-1	-1	0	1	0	0	0	0	0	0	0
BH_2_	1	1	0	-1	0	0	0	0	0	0	0
O_2_	-1	-1	0	0	1	0	0	0	0	0	0
H_2_O	1	1	0	0	0	0	-1	0	0	0	0
H^+^	0	0	-1	-1	0	0	0	0	0	0	1

Rows and columns are labelled, respectively, with the corresponding metabolite and reaction identifiers from Fig 4. The hydrogen ion (H^+^) exchange reaction E7 was omitted from Fig 4 for simplification. The first four columns of *S* correspond to the internal stoichiometric matrix *N* and the last seven columns correspond to the exchange stoichiometric matrix *B*.

**Fig 5:**
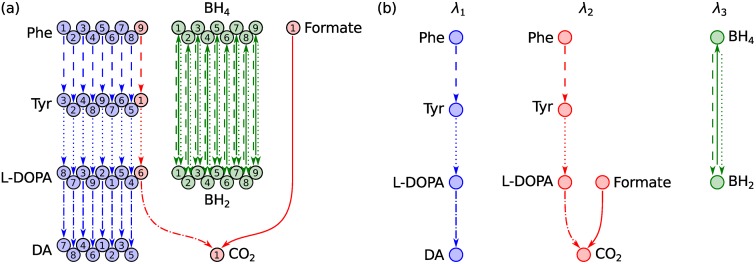
Identification of conserved carbon moieties in DAS. (a) The carbon atom transition network. Numbering of atoms and line styles of atom transitions refer to metabolite structures and reactions, respectively, in Fig 4. The directed graph consists of 18 components, one for each of the nine carbon atoms in L-phenylalanine, and one for each of the nine carbon atoms in tetrahydrobiopterin. The single carbon atom (C1) in formate is in the same component as C9 in L-phenylalanine, since a path can be traced between the two atoms when directionalities of atom transitions are ignored. Isomorphic components have matching colours. A single instance of a conserved moiety consists of all equivalent atoms in a set of isomorphic components. (b) Moiety graphs for the three carbon moieties in DAS. Each graph was obtained by merging a set of isomorphic components in (a) into a single directed graph. Each node represents an instance of a conserved moiety. Each edge represents conservation of a moiety between two metabolites in a particular reaction in Fig 4 with matching line style. Colours match the background colours of the corresponding moieties in Fig 4. Analysis of the full atom transition network for DAS yielded four additional conserved moieties (Fig 6).

To identify all conserved moieties in DAS we require an atom transition network for all atoms regardless of element but for demonstration purposes we will initially focus only on carbon atoms. A carbon atom transition network for DAS is shown in Fig 5a. Our working definition of a conserved moiety is a group of atoms that follow identical paths through a metabolic network. To identify conserved moieties, we therefore need to trace the paths of individual atoms and determine which paths are identical. The paths of individual atoms through the carbon atom transition network for DAS can be traced by visual inspection of Fig 5a. For example, we can trace a path from C1 in L-phenylalanine to C7 in dopamine via C3 in L-tyrosine and C8 in levodopa. This path is made up of atom transitions in reactions R1, R2, and R3 from Fig 4. In graph theory terms, these four carbon atoms and the atom transitions that connect them constitute a *connected component* [25] or, simply, a *component* of the directed graph representing the carbon atom transition network for DAS. A directed graph is said to be *connected* if a path exists between any pair of nodes when edge directions are ignored. A component of a directed graph is a maximal connected subgraph. In total, the carbon atom transition network for DAS in Fig 5a consists of 18 components.

The paths of the first eight carbon atoms (C1–C8) in L-phenylalanine are identical in the sense that they include the same number of atoms in each metabolite and the same number of atom transitions in each reaction. In graph theory terms, the components containing C1–C8 in L-phenylalanine are *isomorphic*. An isomorphism between two graphs is a *structure preserving* vertex bijection [25]. The definition of isomorphism varies for different types of graphs as they have different structural elements that need to be preserved. An isomorphism between two simple graphs is a vertex bijection that preserves the adjacency and nonadjacency of every node, i.e., its connectivity. An isomorphism between two simple directed graphs must also preserve edge directions. We define an isomorphism between two components of an atom transition network as a vertex bijection that preserves the metabolic identity of every node. The nature of chemical reactions ensures that all other structural elements are preserved along with metabolic identities, including the connectivity of atoms and the number, directions and reaction identities of atom transitions. The 18 components of the carbon atom transition network for DAS in Fig 5a can be divided into three sets, where every pair of components within each set is isomorphic.

An isomorphism between two components of an atom transition network is a one-to-one mapping between atoms in the two components. For example, the isomorphism between the two left-most components in Fig 5a maps between C1 and C2 in L-phenylalanine, C3 and C2 in L-tyrosine, C8 and C7 in L-DOPA, and C7 and C8 in dopamine. We say that two atoms are *equivalent* if an isomorphism maps between them. We note that our definition of isomorphism only allows mappings between atoms with the same metabolic identity. Two atoms can therefore only be equivalent if they are in the same metabolite. Equivalent atoms follow identical paths through a metabolic network and therefore belong to the same conserved moiety. In general, *we define a conserved moiety to be a maximal set of equivalent atoms in an atom transition network*. To identify conserved moieties, we must therefore determine isomorphisms between components of an atom transition network to identify maximal sets of equivalent atoms.

**Fig 6:**
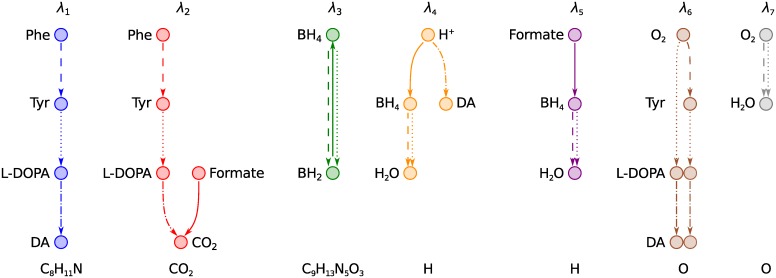
Moiety graphs for all seven conserved moieties in DAS. The seven moieties were identified by analysis of the full atom transition network for DAS in Fig 4. Colours match the background colours of the corresponding moieties in Fig 4. Linestyles of edges match the linestyles of the corresponding reactions in Fig 4. The chemical composition of each moiety is given below its graph.

The first eight carbon atoms (C1–C8) in L-phenylalanine are equivalent. They are therefore part of the same conserved moiety, which we denote *λ*_1_. The last eight carbon atoms (C2–C9) in L-tyrosine are likewise part of the same conserved moiety. They make up another instance of the *λ*_1_ moiety. The *λ*_1_ moiety is conserved between L-phenylalanine and L-tyrosine in reaction R1, between L-tyrosine and levodopa in reaction R2, and between levodopa and dopamine in reaction R3. Each of the four metabolites contains one instance of the *λ*_1_ moiety. The path of this moiety through DAS defines its conservation relation. This brings us to our first representation of moiety conservation relations, which we term moiety graphs. Moiety graphs are obtained from atom transition networks by merging a set of isomorphic components into a single graph. Moiety graphs for the three carbon atom moieties in DAS are shown in Fig 5b. Four additional moieties were identified by analysis of an atom transition network for DAS that included all atoms regardless of element. All seven moiety graphs are shown in Fig 6. Atoms belonging to each node in the moiety graphs are highlighted in Fig 4.

The second way to represent moiety conservation relations is as moiety vectors. Above we defined a moiety vector as a conservation vector *l*_*k*_ where element *l*_*k*,*i*_ corresponds to the number of instances of moiety *k* in metabolite *i* of a metabolic network (see Section Moiety vectors in Theoretical Framework). We can now make this definition exact by relating moiety vectors to moiety graphs. Each instance of a conserved moiety is represented as a node in its moiety graph. Element *l*_*k*,*i*_ of a moiety vector therefore corresponds to the number of nodes in moiety graph λ_*k*_ that represent moieties in metabolite *i*. Moiety vectors are readily derived from moiety graphs by counting the number of nodes in each metabolite. Moiety vectors for DAS were derived from the moiety graphs in Fig 6. The seven moiety vectors are given as columns of the moiety matrix $L\in Z^{11\times 7}$ in Table 4. These seven vectors are linearly independent and therefore span all seven dimensions of $N\;(N^{T})$. The moiety matrix *L* is therefore a *moiety basis* for the left null space.

**Table 4:** Moiety vectors for DAS.

	*l*_1_	*l*_2_	*l*_3_	*l*_4_	*l*_5_	*l*_6_	*l*_7_
Phe	1	1	0	0	0	0	0
Tyr	1	1	0	0	0	1	0
L-DOPA	1	1	0	0	0	2	0
DA	1	0	0	1	0	2	0
CO_2_	0	1	0	0	0	0	0
Formate	0	1	0	0	1	0	0
BH_4_	0	0	1	1	1	0	0
BH_2_	0	0	1	0	0	0	0
O_2_	0	0	0	0	0	1	1
H_2_O	0	0	0	1	1	0	1
H^+^	0	0	0	1	0	0	0

The seven moiety vectors, denoted *l*_1_–*l*_7_ are written as columns of the moiety matrix *L*. Note that *L*_3,6_ = *L*_4,6_ = 2 because levodopa (*i* = 3) and dopamine (*i* = 4) each contain two instances of the *l*_6_ moiety (see moiety graph *λ*_6_ in Fig 6).

### Effects of variable atom mappings between recurring metabolite pairs

Atom transition networks are generated from atom mappings for internal reactions of metabolic networks. However, atom mappings for metabolic reactions are not necessarily unique. Computationally predicted atom mappings, as used here, are always associated with some uncertainty. In addition, there can be biochemical variability in atom mappings, in particular for metabolites containing symmetric atoms. All reactions of the O_2_ molecule, for example, have at least two biochemically equivalent atom mappings since the two symmetric oxygen atoms map with equal probability to connected atoms. Different atom mappings give rise to different atom transition networks that may contain different moiety conservation relations. For the most part, we found that varying the set of input atom mappings did not affect the number of computed moiety conservation relations, only their atomic structure. An important exception was when atom mappings between the same pair of metabolites varied between reactions in the same metabolic network.

**Fig 7:**
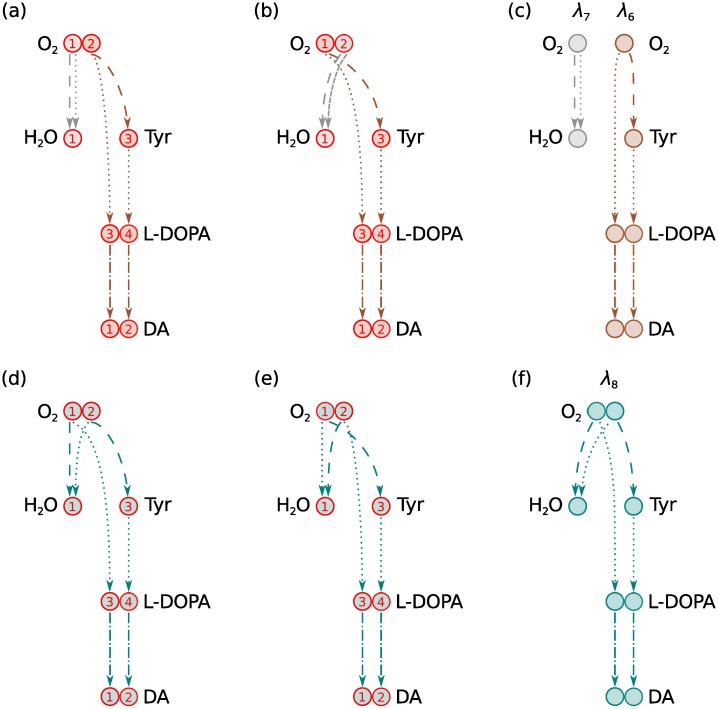
Effects of variable atom mappings between O_2_ and H_2_O in DAS. The recurring metabolite pair exchanges an oxygen atom in two reactions, R1 and R2 in Fig 4. Since the two oxygen atoms of O_2_ are symmetric, there are four possible combinations of oxygen atom mappings for these two reactions. Each combination gives rise to a different oxygen transition network. (a) The first oxygen atom (O1) in O_2_ maps to the single oxygen atom (O1) in H_2_O in both R1 and R2. (b) O2 in O_2_ maps to O1 in H_2_O in both R1 and R2. (c) Moiety graphs obtained from the oxygen atom transition networks in (a) and (b). Two nondecomposable moiety conservation relations were identified in each atom transition network where the same atom mapped from O_2_ to H_2_O in both R1 and R2. (d) O1 in O_2_ maps to O1 in H_2_O in R1 while O2 in O_2_ maps to O1 in H_2_O in R2. (e) O2 in O_2_ maps to O1 in H_2_O in R1 while O1 in O_2_ maps to O1 in H_2_O in R2. (f) The single moiety graph obtained from the oxygen atom transition networks in (d) and (e). Only one composite moiety conservation relation was identified in each atom transition network where a different atom mapped from O_2_ to H_2_O in R1 than R2.

The same pair of metabolites often exchange atoms in multiple reactions throughout the same metabolic network. Common cofactors such as ATP and ADP, for example, exchange atoms in hundreds of reactions in large metabolic networks [26]. In the dopamine synthesis network, DAS in Fig 4, O_2_ and H_2_O exchange an oxygen atom in two reactions, R1 and R2. Since the two oxygen atoms of O_2_ are symmetric, there are four possible combinations of oxygen atom mappings for these two reactions. Each combination gives rise to a different oxygen transition network as shown in Fig 7. Two of these oxygen transition networks, shown in Fig 7a and 7b, contain two moiety conservation relations each, *λ*_6_ and *λ*_7_, which are shown in Fig 7c. The other two oxygen transition networks, shown in Fig 7d and 7e, contain only one moiety conservation relation each, *λ*_8_, which is shown in Fig 7f.

The DAS atom transition network considered in the previous section was generated with the oxygen atom mappings in Fig 7a and thus contained the two moiety conservation relations *λ*_6_ and *λ*_7_ (see Fig 6). An atom transition network generated with the atom mappings in Fig 7d or 7e would contain the single moiety conservation relation *λ*_8_ instead of these two. What distinguishes the oxygen transition networks in Fig 7d and 7e is that the oxygen atom in O_2_ that maps to H_2_O varies between the two reactions R1 and R2. The atom transition network for DAS therefore contains one less moiety conservation relation if the atom mapping between this recurring metabolite pair varies between reactions. The moiety matrix for these alternative atom transition networks,

$$L=\left[l_{1},l_{2},l_{3},l_{4},l_{5},l_{8}\right],$$ (5)

only contains six linearly independent columns and is therefore not a basis for the seven dimensional left null space of *N*.

The vector representation of moiety graph *λ*_8_ is

$$l_{8}^{T}=\left[\begin{array}{ccccccccccc} 0 & 1 & 2 & 2 & 0 & 0 & 0 & 0 & 2 & 1 & 0 \end{array}\right].$$ (6)

We note that *l*_8_ = *l*_6_ + *l*_7_ where

$$l_{6}^{T}=\left[\begin{array}{ccccccccccc} 0 & 1 & 2 & 2 & 0 & 0 & 0 & 0 & 1 & 0 & 0 \end{array}\right],$$ (7)

$$l_{7}^{T}=\left[\begin{array}{ccccccccccc} 0 & 0 & 0 & 0 & 0 & 0 & 0 & 0 & 1 & 1 & 0 \end{array}\right],$$ (8)

from Table 4. The moiety vector *l*_8_ therefore represents a composite moiety. It does not meet the definition of a nondecomposable moiety vector in Eq 4. This example shows that variable atom mappings between recurring metabolite pairs may cause multiple nondecomposable moiety conservation relations to be joined together into a single composite moiety conservation relation. We formulated an optimisation problem, described in Methods, Section Decomposition of moiety vectors, to decompose composite moiety vectors. Solving this problem for the composite moiety vector *l*_8_ yields the two nondecomposable components *l*_6_ and *l*_7_.

### General properties of identified moieties

We applied our method to identify conserved moieties in three metabolic networks of increasing size. The networks, listed from smallest to largest, were the dopamine synthesis network, DAS in Fig 4, the *E. coli* core metabolic network, iCore [27], and an atom mapped subset of the generic human metabolic reconstruction, Recon 2 [26] which we refer to here as subRecon. The dimensions of the three networks are given in Table 5a. Further descriptions are provided in Methods, Section Metabolic networks. There are seven linearly independent conservation relations for the closed DAS network, 11 for iCore, and 351 for subRecon.

**Table 5:** Results for the three metabolic networks treated here.

(a)			
Network	DAS	iCore	subRecon
Metabolites (*m*)	11	72	2,970
Internal reactions (*u*)	4	74	4,261
rank(*N*)	4	61	2,619
dim(null(*N*^*T*^))	7	11	351
(b)			
Initial moieties (*r*)	7	10	345
rank(*L*)	7	10	340
Decomposed moieties (*t*)	7	11	353
rank(*D*)	7	11	347
(c)			
Carbon atom isotopomers	2.8×10^3^	1.1×10^15^	6.2×10^23^
Carbon moiety isotopomers	2.2×10^1^	1.4×10^3^	4.9×10^18^
(d)			
Atoms (*p*)	170	1,697	153,298
Atom transitions (*q*)	176	6,019	446,900
Graph-based method (this work)	1.8×10^-1^	5.6×10^-1^	2.8×10^2^
Vertex enumeration algorithm [13]	6.1×10^-2^	9.1×10^-1^	>6.0×10^5^

(a) Dimensions of stoichiometric matrices. The number of linearly independent conservation relations is $\text{dim}(N(N^{T}))=m-\text{rank}(N)$ in a closed network with stoichiometric matrix $N\in Z^{m\times u}$. (b) Dimensions of moiety matrices. Initial moiety matrices $L\in N_{0}^{m\times r}$ were computed directly from predicted atom transition networks. Decomposed moiety matrices $D\in N_{0}^{m\times t}$ were derived by decomposing the columns of *L* as described in Methods, Section Decomposition of moiety vectors. (c) Carbon isotopomers (see Section Application of moiety graphs to stable isotope assisted metabolic flux analysis). Comparison between the number of carbon atom and carbon moiety isotopomers. (d) Computation times (in seconds) for the graph-based method presented here, in comparison to the vertex enumeration algorithm described in [13] (see Section Computational complexity).

**Fig 8:**
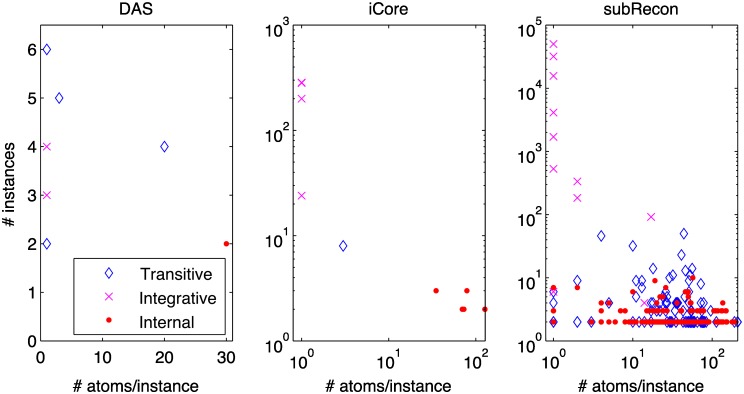
Characteristics of conserved moieties identified in the three metabolic networks treated here. The total number of instances of a moiety is plotted against the number of atoms per instance. Classification of moieties as transitive, internal, or integrative is described in, Methods, Section Classification of moieties.

Atom transition networks were generated using algorithmically predicted atom mappings [20] as described in Methods, Section Generation of atom transition networks. Seven, ten and 345 moiety conservation relations were identified in the predicted atom transition network for DAS, iCore and subRecon, respectively (Table 5b). Characterisation of identified moieties revealed some trends (Fig 8). We found a roughly inverse relationship between the frequency of a moiety, defined as the number of instances, and the size of that moiety, defined as the number of atoms per instance. We also found a relationship between moiety size, frequency and classification. Internal moieties tended to be large and infrequent, occurring in a small number of closely related secondary metabolites, e.g., the 35 atom AMP moiety found in the three iCore metabolites AMP, ADP and ATP. Integrative moieties were usually small and frequent while transitive moieties were intermediate in both size and frequency. The smallest moieties consisted of single atoms. These were often highly frequent, occurring in up to 62/72 iCore metabolites and 2,472/2,970 subRecon metabolites. These results indicate a remarkable interconnectivity between metabolites at the atomic level. Due to their frequency, single atom moieties accounted for a large portion of atoms in each metabolic network. Single atom moieties accounted for nearly half (791/1,697) of all atoms in iCore, and approximately two thirds (104,268/153,298) of all atoms in subRecon.

Moiety matrices derived from the predicted atom transition networks for iCore and subRecon did not span the left null spaces of their respective stoichiometric matrices, indicating that they might contain composite moiety vectors. Using the method described in Methods, Section Decomposition of moiety vectors, we found two composite moiety vectors in the moiety matrix for iCore, and 10 in the one for subRecon. Decomposition of these vectors yielded three new nondecomposable moiety vectors for iCore and 18 for subRecon (Table 5b). The 11 nondecomposable moiety vectors for iCore were linearly independent. They therefore comprised a basis for the 11 dimensional left null space of *N* for iCore. The 353 nondecomposable moiety vectors for subRecon, on the other hand, were not linearly independent and only spanned 347 out of 351 dimensions in the left null space of *S* for subRecon. This indicated that there existed conservation relations for subRecon that were independent of atom conservation.

Schuster and Höfer, citing earlier work by Aris [28] and Corio [29], noted the importance of considering electron conservation in addition to atom conservation [11]. Unfortunately, it is not as straightforward to map electrons as atoms and no formalism currently exists for electron mappings. As a result, electron conservation relations cannot be computed with the current version of our algorithm. We therefore computed electron conservation relations for subRecon by decomposing the electron vector with the method described in Methods, Section Decomposition of moiety vectors. An electron vector for a metabolic network with m metabolites is a vector $e\in N^{m}$ where *e*_*i*_ is the total number of electrons in metabolite *i*. Decomposition of *e* for subRecon yielded 11 new conservation vectors. When combined, the 11 electron vectors and the 353 fully decomposed moiety vectors for subRecon (Table 5b) spanned the left null space of the subRecon stoichiometric matrix.

### The gearwheels of metabolism

Internal moieties define pools of metabolites with constant total concentration and dependent individual concentrations. In the small dopamine synthesis network DAS in Fig 4, the biopterin moiety (*l*_3_) is classified as internal. This moiety is conserved between the metabolites BH_2_ and BH_4_. The total concentration of BH_2_ and BH_4_ is therefore fixed at a constant value in DAS. If the concentration of BH_2_ increases, the concentration of BH_4_ must decrease by the same amount and vice versa.

The concentration dependency between BH_2_ and BH_4_ couples all reactions that interconvert the two metabolites. Assume that DAS is initially at a steady state when there is a sudden increase in flux through reactions R1, R2, R3 and associated exchanges such that the concentrations of all primary metabolites remain constant. This would lead to net consumption of BH_4_ accompanied by net production of BH_2_. The increased BH_2_/BH_4_ concentration ratio would increase thermodynamic and mass action kinetic driving forces through R4, while simultaneously decreasing driving forces through R1 and R2. The system would eventually settle back to the initial steady state or a new one depending on reaction kinetic parameters and substrate availability. Conservation of the biopterin moiety therefore imposes a purely physicochemical form of regulation on dopamine synthesis that is mediated through mass action kinetics and thermodynamics. This statement can be generalised to all internal moieties, as Reich and Sel’kov did in their 1981 monograph on energy metabolism [2].

**Fig 9:**
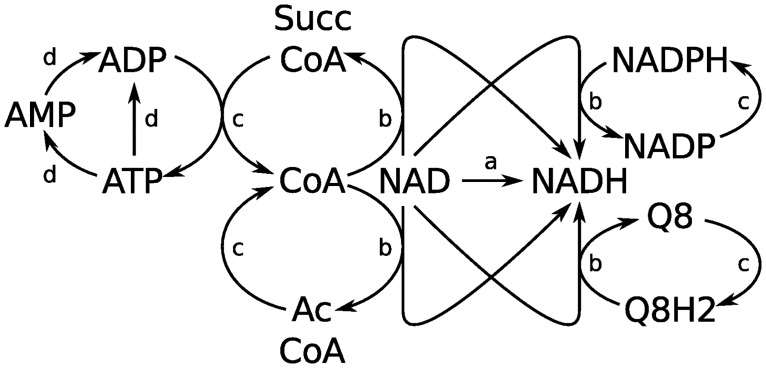
Coupling between internal moiety pools in iCore. The five pools from Table 6 are coupled into a gearwheel-like mechanism. An increase in the NAD/NADH concentration ratio would affect driving forces in the direction shown. (a) Any reactions that interconvert NAD and NADH would be driven in the direction of increased NAD consumption. These include reactions of glycolysis and the TCA cycle, reactions converting malate and lactate to pyruvate, and reactions converting pyruvate, ethanol, and acetaldehyde to acetyl CoA. In short, NAD/NADH coupled reactions would be driven in the direction of increased acetyl CoA production from available carbon sources. (b) The increased NAD/NADH concentration ratio would also affect driving forces through reactions that couple the NAD pool to other cofactor pools. Altered flux through these reactions would in turn affect concentration ratios within those pools which are coupled to their own sets of reactions. (c) An increased NADP/NADPH ratio would drive flux through the pentose phosphate pathway and conversion of glutamate to alpha-ketoglutarate. An increased Q8/Q8H2 ratio would inhibit flux through the electron transport chain. Increased acetyl-CoA/CoA and succinyl-CoA/CoA ratios would drive acetate production and TCA cycle reactions, respectively, which are coupled to ATP production from ADP. (d) An increase in the ATP/ADP ratio resulting from increased flux through these reactions would drive ATP consuming reactions. In iCore, ATP consuming reactions are mainly found in gluconeogenesis so the increased ATP/ADP ratio would counteract the effects of an increased NAD/NADH ratio to some extent.

Reich and Sel‘kov’s gearwheel analogy [2] is appropriate for the five internal moieties we identified in iCore. These five moieties define five well known cofactor pools (Table 6). Each pool is coupled to a set of reactions that interconvert metabolites within that pool. The five pools are also coupled to each other through shared reactions, forming a gearwheel-like mechanism (Fig 9). A change in concentration ratios within any pool will affect the driving forces that turn the wheels. The central wheel in iCore is the NAD moiety (*l*_6_). A change in concentration ratios within one pool will therefore be propagated to other pools via the NAD/NADH concentration ratio (Fig 9). This example shows how local changes in the state of a metabolic network can be propagated throughout the network via coupled cofactor pools defined by internal moieties.

**Table 6:** Internal moieties in iCore.

Moiety	Chemical composition	Metabolites
*l*_1_	C_49_H_74_O_4_	Q8, Q8H2
*l*_2_	C_21_H_25_N_7_O_17_P_3_	NADP, NADPH
*l*_4_	C_10_H_12_N_5_O_7_P	AMP, ADP, ATP
*l*_6_	C_21_H_26_N_7_O_14_P_2_	NAD, NADH
*l*_7_	C_21_H_31_N_7_O_16_P_3_S	CoA, Acetyl-CoA, Succinyl-CoA

The majority of moieties identified in subRecon were classified as internal (237/345). Most of these internal moieties were artefacts of the way the subset of reactions from Recon 2 were selected, i.e., based on the availability of atom mapping data (see Methods, Section Metabolic networks). Many reactions in subRecon were disconnected from the rest of the network and therefore could not carry any flux. To identify reactions capable of carrying flux, we computed the flux consistent part of subRecon [30], which consisted of 3,225 reactions and 1,746 metabolites. We identified 118 moiety conservation relations for this part of subRecon, 33 of which were classified as internal. The metabolite pools defined by these moieties consisted of between 2 and 9 metabolites and were distributed across five cell compartments; the cytosol, mitochondria, nucleus, endoplasmic reticulum, and peroxisomes. Some moieties were compartment specific while others were distributed amongst metabolites in two different compartments. As in iCore, the internal moiety pools were not independent of each other but were coupled by shared reactions.

### Application of moiety graphs to stable isotope assisted metabolic flux analysis

Atoms in the same instance of a conserved moiety all follow the same path through a metabolic network. In an atom transition network these atoms are represented as separate nodes and their atom transitions as separate edges. A moiety graph encodes the paths of all atoms in an atom transition network in a reduced number of nodes and edges. In effect, they are reduced representations of atom transition networks that can be used in many of the same applications. Atom transition networks arise most frequently in the context of stable isotope assisted metabolic flux analysis where they underpin the ability to model the flow of isotopically labelled atoms through metabolic networks [31]. Stable isotope assisted metabolic flux analysis (MFA) deals with estimation of internal reaction fluxes in a metabolic network based on data from isotope labelling experiments [31]. Internal fluxes are estimated by fitting a mathematical model to measured exchange fluxes and isotopomer distributions.

A basic MFA model consists of nonlinear flux balance equations formulated around isotopomers of metabolites in the metabolic network of interest [32]. A metabolite with *n* carbon atoms has 2^*n*^ carbon atom isotopomers. Therefore, the number of isotopomer balance equations grows exponentially with the number of metabolites in the metabolic network. More sophisticated MFA modelling frameworks have been developed to reduce the complexity of the problem, notably the cumomer [33] and elementary metabolite unit (EMU) [34] frameworks. Cumomer models consist of flux balance equations formulated around transformed variables called cumomers. They are the same size as isotopomer models but have a simpler structure that makes them easier to solve. EMU models have a similar structure as cumomer models but are significantly smaller. They consist of flux balance equations formulated around transformed variables known as EMU species. The number of EMU species for a given metabolic network is much smaller than the number of isotopomers and cumomers.

MFA models can be derived from moiety graphs instead of atom transition networks without loss of predictive capacity. We say that a moiety is labelled if any of its atoms are labelled and define moiety isotopomers as different labelling states of a metabolite’s moieties. The eight carbon containing metabolites in DAS (Fig 4) have 2,820 possible carbon atom isotopomers. Their 55 carbon atoms can be grouped into 11 carbon moieties (Fig 5b) with only 22 possible carbon moiety isotopomers. The reduction in number of isotopomers is even more pronounced for the two larger metabolic networks (Table 5c), reaching 12 orders of magnitude for iCore. It was less for subRecon where a greater proportion of moieties consist of a single atom (Fig 8). However, it was still substantial. Deriving MFA models from moiety graphs can therefore reduce the number of model equations by several orders of magnitude. Isotopomer and cumomer models, in particular, can be simplified with this approach. The algorithm to generate EMU species from atom transition networks ensures that atoms in the same instance of a conserved moiety are always part of the same EMU species. EMU models derived from moiety graphs will therefore be identical to those derived from atom transition networks (see supporting file S1 Fig). Regardless of the MFA modelling framework, moiety graphs can be used to simplify design of isotope labelling experiments, by reducing the number of options for labelled substrates.

### Application of moiety vectors to decomposition of metabolic networks

Moiety vectors can be used to decompose a metabolic network into simpler moiety subnetworks [35]. An open metabolic network with total stoichiometric matrix *S* can be decomposed into *t* moiety subnetworks where *t* is the number of moiety conservation relations for the corresponding closed network *N*. Each moiety vector $l_{k}\in N\;(N)$ defines a stoichiometric matrix for one moiety subnetwork as

$$S^{\left(k\right)}=diag\left(l_{k}\right)S.$$ (9)

**Fig 10:**
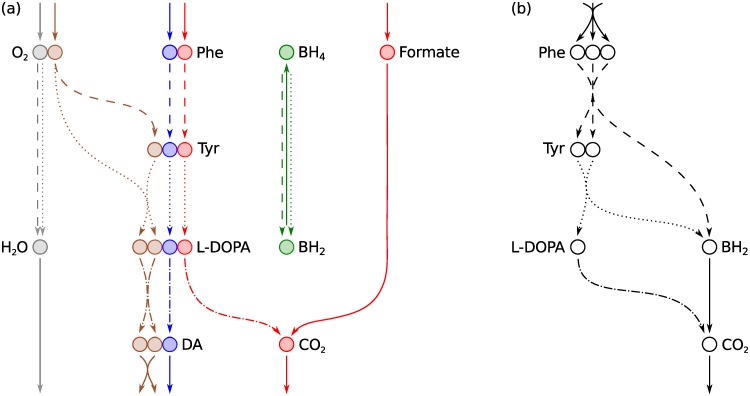
Moiety subnetworks of DAS. (a) Moiety vectors *l*_1_, *l*_2_, *l*_3_, *l*_6_, and *l*_7_ (Table 4) were used to decompose the stoichiometric matrix for DAS (Table 3) into five subnetworks. Colours match the corresponding moieties in Fig 4. Linestyles match the corresponding reactions in Fig 4. The two hydrogen atom moiety subnetworks (*l*_4_ and *l*_5_) were omitted to simplify the figure. (b) A subnetwork derived from an extreme ray that did not represent moiety conservation. This subnetwork is not mass balanced as there is no mass transfer between Phe and BH_2_, Tyr and BH_2_, or BH_2_ and CO_2_ in the full metabolic network (Fig 4).

Stoichiometric matrices for moiety subnetworks (*S*^(*k*)^) are generally more sparse than the stoichiometric matrix for the full metabolic network (*S*). Each moiety subnetwork only includes the subparts of metabolites and reactions that involve a particular moiety. Moiety subnetworks of DAS are shown in Fig 10a. In addition to being more sparse than the full metabolic network (Fig 4), these subnetworks have simpler topologies. Of the seven moiety subnetworks of DAS only one (*S*^(6)^) was a hypergraph. All other DAS subnetworks were graphs. Four of 11 iCore subnetworks and 342 of 365 subRecon subnetworks were also graphs. We note that, although metabolic networks could in theory be decomposed with other types of conservation vectors, only moiety vectors are guaranteed to result in mass balanced subnetworks (see Fig 10b).

### Instantaneous moieties

The results above were for moieties identified for metabolic network reconstructions where we assume each reaction is active. These moieties will only be relevant if all reactions in those reconstructions are actually active in practice, i.e., carrying nonzero flux. In general, not all reactions in a metabolic network are active simultaneously, e.g., oxidative phosphorylation reactions in iCore are only active in the presence of oxygen. The set of instantaneous conserved moieties, their conservation relations, and their classification depend on which reactions are active at any point in time. All steady state flux distributions $v\in R^{n}$ are in the right null space N(S) of the total stoichiometric matrix *S* for a metabolic network [36]. A convex basis for N(S) gives all extreme pathways of a metabolic network [37]. Extreme pathways are analogous to extreme semipositive conservation relations in the left null space $N(S^{T})$ (see Section Introduction). They are a maximal set of conically independent steady state flux distributions. Any steady state flux distribution can be written as a conical combination of extreme pathways.

**Fig 11:**
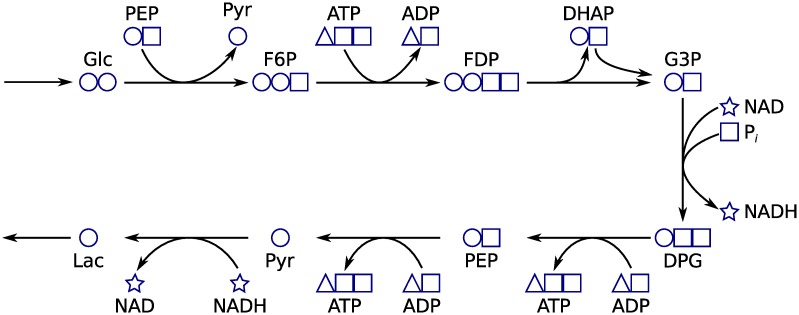
Instantaneous iCore moieties. Carbon and phosphate containing moieties in an extreme pathway of the *E. coli* core network that corresponds to glycolysis. Four conserved moieties are distinguished by shape in the figure. The pathway also conserves one oxygen atom moiety and two hydrogen atom moieties that were omitted to simplify the figure. Metabolite abbreviations are, Glc: D-glucose (VMH [23] ID: glc_D), PEP: phosphoenolpyruvate (VMH ID: pep), Pyr: pyruvate (VMH ID: pyr), F6P: D-fructose 6-phosphate (VMH ID: f6p), ATP: adenosine triphosphate (VMH ID: atp), ADP: adenosine diphosphate (VMH ID: adp), FDP: D-fructose 1,6-bisphosphate (VMH ID: fdp), DHAP: dihydroxyacetone phosphate (VMH ID: dhap), G3P: glyceraldehyde 3-phosphate (VHM ID: g3p), NAD: nicotinamide adenine dinucleotide (VMH ID: nad), P_*bluei*_: orthophosphate (VMH ID: pi), NADH: reduced nicotinamide adenine dinucleotide (VMH ID: nadh), DPG: 1,3-bisphospho-D-glycerate (VMH ID: 13dpg), Lac: D-lactate (VMH ID: lac_D). The glucose moiety (circles) is transitive whereas the other three moieties are internal, including the phosphate moiety (squares) which was classified as integrative in the full iCore network.

To see how instantaneous conserved moieties vary depending on what reactions are active we computed the extreme pathways of iCore with the vertex enumeration algorithm from [13]. Computation of the extreme pathways of subRecon with the same algorithm was not tractable. The algorithm returned 1,421 extreme pathways for iCore. The number of instantaneous moiety conservation relations for these pathways ranged from 4 to 11 and the total number of moieties (i.e., instances) ranged from 18 to 520. Fig 11 shows an example of instantaneous moieties in an extreme pathway that corresponds to glycolysis. We found that moieties classified as transitive or integrative in the entire iCore network, were often classified as internal in individual extreme pathways. In particular, the inorganic phosphate moiety (P_*i*_) was classified as internal in all except one extreme pathway. The constant metabolite pool defined by the P_*i*_ moiety varied between pathways, consisting of P_*i*_, ATP, AMP and 9 to 17 phosphorylated intermediates of glycolysis and the pentose phosphate pathway. The ammonia moiety (NH_4_^+^) was also classified as internal in many extreme pathways (266/1,421) where it defined a constant metabolite pool consisting of NH_4_^+^, glutamine and glutamate.

### Computational complexity

The computational complexity of the method presented here is largely determined by the following two steps: 1) finding connected components of an atom transition network, and 2) determining isomorphisms between components. We used an implementation of Tarjan’s Algorithm [38] to find connected components of atom transition networks (see Methods, Section Identification of conserved moieties). The worst case time complexity of this algorithm is *O* (*p* + *q*) where *p* is the number of atoms (nodes) and *q* is the number of atom transitions (edges) in the input atom transition network. We apply Tarjan’s algorithm to the simple graph underlying the input atom transition network, which generally contains significantly fewer edges.

Algorithms to determine isomorphisms between two general graphs are an active research area. Atom transition networks are specialised graphs where every node is associated with a metabolite and every edge is associated with a reaction in the parent metabolic network. These additional structural elements of atom transition networks make it possible to determine isomorphisms between their components by pairwise comparisons (see Section Identification of conserved moieties in Methods). Since every atom must be connected to at least one other atom, the number of components is bounded from above by *p*/2. The number of components in the atom transition networks treated here was much lower. There were 57 components in the atom transition for DAS, 391 in the one for iCore, and 16,348 in the one for subRecon. If no component is isomorphic to any other component, we need to compare the first component to all other components, the second component to all others except the first, etc. The maximum number of comparisons is therefore

$$\left(\frac{p}{2}-1\right)+\left(\frac{p}{2}-2\right)+\cdots +\left(\frac{p}{2}-\frac{p}{2}\right)=\frac{p^{2}}{4}-\sum_{g=1}^{p/2}g=\frac{p^{2}}{4}-\frac{1}{2}\left(\frac{p^{2}}{4}+\frac{p}{2}\right)=\frac{1}{4}\left(\frac{p^{2}}{2}-p\right).$$ (10)

The overall worst case time complexity of our method is therefore $O(p^{2}+q)$. In practice, however, computation time scales much better (Table 5d). Identification of conserved moieties in subRecon took under five minutes with our method. We compared this performance with an implementation of a vertex enumeration algorithm [13] to compute the extreme rays of the left null space of a stoichiometric matrix (Table 5d). The two algorithms performed similarly on the two smaller networks but computation of extreme rays proved intractable for subRecon. The vertex enumeration algorithm did not complete after running for a week, at which point we terminated the process.

It may be of interest to know how our method scales with the size of metabolic networks, instead of the size of atom transition networks. The number of atoms per metabolite varies greatly but is bounded from above. So is the number of atom transitions per reaction. The largest metabolite in the three metabolic networks treated here was the subRecon metabolite neurotensin (Recon 2 ID C01836), with 241 atoms. The largest reaction was the subRecon reaction peroxisomal thiolase 2 (Recon 2 ID SCP2x), with 1,791 atom transitions. This is a composite reaction with large stoichiometric coefficients. Such large reactions are anomalous. The average number of atom transitions per metabolic reaction was much lower. The average (±standard deviation) was 44 (±16) for DAS, 81 (±72) for iCore, and 105 (±90) for subRecon. The number of atoms and atom transitions scales approximately linearly with the number of metabolites and internal reactions, respectively (Table 5d). We can therefore approximate the worst case time complexity of our method as $O(m^{2}+u)$.

## Discussion

Moiety conservation relations are a subset of nonnegative integer conservation relations for a metabolic network. In principle, the latter can be computed using only a stoichiometric matrix, but the computational complexity of existing algorithms [11, 12, 14, 15, 17] has prohibited their application to large networks. Computation of moiety conservation relations requires information about the paths of atoms through metabolic networks in addition to reaction stoichiometry (see, Section Theoretical Framework, Section Moiety vectors). Here, we incorporated this information in the form of atom transition networks. Doing so allowed us to formulate the problem of computing moiety conservation relations as a graph theory problem that is solvable in polynomial time. We related atom paths to connected components of atom transition networks and conserved moieties to equivalent nodes of isomorphic components. We provided a novel definition of isomorphism that is specific to the structure of atom transition networks. This definition enabled us to determine isomorphisms and identify conserved moieties in a fast and reliable way. The relationship between conservation relations and metabolite substructures has long been known [1, 2, 18]. A relationship between conservation relations and graph theoretical properties of atom transition networks has not, to our knowledge, been demonstrated prior to this work. This is also, to our knowledge, the first polynomial time method to compute nonnegative integer conservation relations for metabolic networks.

Our method requires data on reaction stoichiometry and atom mappings for internal reactions of a metabolic network. Reliable data on reaction stoichiometry are readily available from high quality, manually curated metabolic network reconstructions that have been published for hundreds of organisms over the past couple of decades or so. These reconstructions are accessible in a standardised format [39], e.g., through the BioModels database [40]. Atom mapping data are increasingly becoming accessible through biochemical databases but are still largely algorithmically generated [21, 22]. KEGG [41, 42] and BioPath (Molecular Networks GmbH, Erlangen, Germany) provide manually curated atom mappings but the data are not freely accessible. No database currently provides mappings for hydrogen atoms or electrons which are required to compute all conserved moieties in a metabolic network. Data formats vary between databases as there is currently no agreed standard. However, the availability and quality of atom mapping data are rapidly increasing and we expect these issues will be remedied in the near future.

We chose to use the DREAM algorithm [20] to predict atom mappings for this work. Advantages of DREAM include ease of use, the ability to map hydrogen atoms, and use of the information-rich rxnfile format. A disadvantage of DREAM is that it uses mixed integer linear programming (MILP) which has exponential worst case time complexity. Kumar and Maranas recently published the first polynomial time atom mapping algorithm, called canonical labelling for clique approximation (CLCA) [22]. An implementation of this algorithm has not yet been released but should further speed up the process of obtaining atom mapping predictions. CLCA predictions for 27,000 reactions are already accessible through the MetRxn database [22]. These predictions were not yet suitable for this work, however, as they do not include hydrogen atoms.

Conserved moieties identified with our method depend on input atom mappings (see Results, Section Effects of variable atom mappings between recurring metabolite pairs). We showed how variable atom mappings between recurring metabolite pairs could give rise to a non-maximal set of composite moiety vectors. Note that composite moieties are a biochemical reality, not just an artefact of the atom mapping algorithm used. Many metabolite pairs do have multiple biochemically equivalent atom mappings, each of which is realised in a living organism. For modelling purposes, however, it is desirable to identify a maximal number of linearly independent moiety conservation relations. We therefore formulated an MILP algorithm for decomposition of composite moiety vectors (Methods, Section Decomposition of moiety vectors). It would be preferable to construct the atom transition network with minimal variability in atom mappings between recurring metabolite pairs to avoid composite moieties altogether. Doing so would be relatively straightforward if input data included all alternative atom mappings for reactions. Prediction of alternative atom mappings with the DREAM algorithm is possible but time consuming, both due to the longer running times required, and because DREAM outputs each alternative atom mapping in a separate rxnfile. Some effort is therefore required to integrate alternative predictions. The CLCA algorithm outputs alternative atom mapping predictions in a single file by default and should therefore facilitate identification of nondecomposable moiety conservation relations. Ultimately, however, predicted atom mappings need to be manually curated for alternatives.

To span the left null space of Recon 2 we needed to decompose the electron vector $e\in N_{0}^{m}$ (Results, Section General properties of identified moieties) with the MILP algorithm described in Methods, Section Decomposition of moiety vectors. We note that this MILP algorithm can also be used to decompose the elemental matrix for a metabolic network. This is in fact a method for nonnegative integer factorisation of the elemental matrix, similar to the algorithm presented in [18]. However, this method has exponential worst case time complexity. Also, while MILP decomposition of the elemental matrix returns the chemical composition of moieties it cannot be used to pinpoint the exact group of atoms in a metabolite that belong to each moiety. Empirically, we found that MILP decomposition of the elemental matrices for the three metabolic networks treated here completed in a reasonable amount of time although it scaled much worse than analysis of atom transition networks (3.4 × 10^−1^ s for DAS, 1.8 × 10^0^ s for iCore, 4.7 × 10^3^ s for subRecon, compare to Table 5d). In the absence of atom mapping data, MILP decomposition of the elemental matrix provides an alternative way to compute moiety conservation relations for metabolic networks. For the most part, decomposition of elemental matrices gave the same set of vectors as analysis of atom transition networks. The only exception was that decomposition of the elemental matrix for DAS returned the vector

$$l_{9}^{T}=\left[\begin{array}{ccccccccccc} 0 & 1 & 2 & 0 & 2 & 2 & 0 & 0 & 1 & 0 & 0 \end{array}\right],$$ (11)

in place of the oxygen moiety vector *l*_6_ in Table 4. We note that *l*_9_ = *l*_6_ + 2 (*l*_2_ − *l*_1_) does not correspond to a conserved moiety in DAS.

Here, we highlighted three potential applications of our method; to identify constant metabolite pools (Results Section The gearwheels of metabolism), to model isotope labelling experiments for metabolic flux analysis (Results Section Application of moiety graphs to stable isotope assisted metabolic flux analysis), and to decompose metabolic networks (Results Section Application of moiety vectors to decomposition of metabolic networks). These applications take advantage of our method’s unique ability to identify the exact group of atoms that correspond to each conserved moiety. As we alluded to in the introduction, another clear application area is metabolic modelling. A nonnegative integer basis for the left null space can be used to simplify metabolic models and to compute a full rank Jacobian which is required for many computational modelling methods [6, 7]. Other applications would include minimisation of intermediate metabolite concentrations [43], and computation of minimal cut sets [44]. We also believe our method may be of value to theoretical biologists. For example, the ability to decompose metabolic networks into simpler subnetworks may facilitate research on physical and mathematical properties that are otherwise obscured by topological complexity.

## Methods

### Metabolic networks

We tested our method on three metabolic networks of increasing sizes (see Table 5a), two human and one *E. coli* network. The *E. coli* network consisted of core metabolic pathways including glycolysis, the pentose phosphate shunt, the TCA cycle, oxidative phosphorylation and fermentation [27]. We refer to this network as iCore for abbreviation.

The two human networks were derived from the generic human metabolic reconstruction Recon 2 [26]. The smaller of the two consisted of four internal reactions from the dopamine synthesis pathway and seven metabolite exchange reactions. We refer to this network as DAS, and its four internal reactions as R1, R2, R3, and R4. R1 corresponds to Recon 2 reaction r0399, R2 is a composite of reactions TYR3MO2 and THBPT4ACAMDASE, R3 corresponds to reaction 3HLYTCL, and R4 is a composite of reactions DHPR and FDH.

The larger human network, which we refer to as subRecon, included approximately two thirds (4,261/6,691) of internal reactions in Recon 2. This was the largest subset of Recon 2 reactions for which atom mappings could be predicted at the time of our analysis. For most of the remaining reactions (2,380/2,430), we were unable to generate rxnfiles for input to the DREAM server [20]. For other reactions (50/2,430), the DREAM algorithm timed out or failed to parse input rxnfiles. Rxnfiles could not be generated for 1,871/2,380 due to lack of information about metabolite structures, and for 509/2,380 reactions because they were not mass or charge balanced.

### Generation of atom transition networks

Atom transition networks were generated based on atom mappings for metabolic reactions. Atom mapping predictions were obtained through the web interface to the mixed integer linear programming method DREAM [20]. The objective was set to minimise the number of bonds broken and formed in each reaction. Reactions were input to DREAM in rxnfile format (Accelrys, San Diego, CA). Rxnfiles were written from data on reaction stoichiometry and metabolite structures in molfile format (Accelrys, San Diego, CA). All hydrogen atoms were explicitly represented to obtain mappings for hydrogen atoms in addition to other elements. Care was taken to ensure that hydrogen and charge balancing of reactions was the same in rxnfiles as in the parent stoichiometric matrix. This was essential to ensure that computed moiety vectors were in the left null space of the stoichiometric matrix.

### Identification of conserved moieties

We denote the internal stoichiometric matrix of a metabolic network by $N\in Z^{m\times u}$. Conserved moieties in the metabolic network were identified by analysis of an atom transition network that was generated as described in Generation of atom transition networks. We denote the incidence matrix of the input atom transition network by *A* ∈ {−1, 0, 1}^*p*×*q*^ where *p* is the number of atoms and *q* the number of atom transitions. The first step in our analysis is to find connected components of *A*. To this end, we used an implementation of Tarjan’s algorithm [38] (see Section Implementation). We denote the incidence matrix of component *h* of *A* by *C*^(*h*)^ ∈ {−1, 0, 1}^*x*×*y*^.

Each atom in a component belongs to a particular metabolite in the metabolic network. We define a mapping matrix *M*^(*h*)^ ∈ {0, 1}^*m*×*x*^ that maps atoms to metabolites. It is defined such that $M_{i,g}^{\left(h\right)}=1$ if the atom represented by row *g* in *C*^(*h*)^ belongs to the metabolite represented by row *i* in *N*. Otherwise, $M_{i,g}^{\left(h\right)}=0$. The component *C*^(*h*)^ represents conservation of a single atom throughout the metabolic network. We define its atom conservation vector as

$$a_{h}=M^{\left(h\right)}1,$$ (12)

i.e., it is the column sum of *M*^(*h*)^. Element *a*_*h*,*i*_ is therefore the number of atoms in metabolite *i* that are in component *C*^(*h*)^. We define two components *C*^(*h*)^ and *C*^(*d*)^ to be isomorphic if they include the same number of atoms from each metabolite. It follows that the two components are isomorphic, with *C*^(*h*)^ = *C*^(*d*)^, if *a*_*h*_ = *a*_*d*_. A set of isomorphic components is denoted by *K* = {*h*, *d* ∣ *a*_*d*_ = *a*_*h*_}.

A moiety graph *λ*_*k*_ is obtained by merging a set *K* of isomorphic components into a single graph. The incidence matrix of *λ*_*k*_ is given by

$$G^{\left(k\right)}=\frac{1}{\left|K\right|}\sum_{h\in K}C^{\left(h\right)}.$$ (13)

We note that *G*^(*k*)^ = *C*^(*h*)^∀*h* ∈ *K* except that the rows of *G*^(*k*)^ represent separate instances of a conserved moiety instead of atoms. A moiety vector *l*_*k*_ is derived from the incidence matrix *G*^(*k*)^ of a moiety graph in the same way that the atom conservation vector *a*_*h*_ was derived from the incidence matrix *C*^(*h*)^ of a component in Eq 12. This is equivalent to setting *l*_*k*_ = *a*_*h*_∀*h* ∈ *K*.

### Classification of moieties

We classified moieties according to the schema presented in [12]. Briefly, moieties were grouped into three categories termed transitive, integrative and internal. These categories were referred to as Type A, Type B, and Type C, respectively, in [12]. A moiety with conservation vector *l*_*k*_ was classified as internal if it was conserved in the open metabolic network represented by the total stoichiometric matrix *S*, i.e., if *S*^*T*^ *l*_*k*_ = 0. Metabolites containing internal moieties were defined as secondary metabolites, while all other metabolites were defined as primary metabolites. Moieties that were only found in primary metabolites were classified as transitive moieties, while those that were found in both primary and secondary metabolites were classified as integrative moieties.

### Decomposition of moiety vectors

Our method for analysing atom transition networks returns *r* moiety vectors $\left\{l_{k}\in N_{0}^{m}|k\in [1,r]\right\}$ as the columns of the moiety matrix $L\in N_{0}^{m\times r}$. As described in Results, Section Effects of variable atom mappings between recurring metabolite pairs, our method may return composite moiety vectors if the input atom transition network was generated from variable atom mappings between recurring metabolite pairs. Any composite moiety vector can be written as *l_k_* = *x_k_* + *y_k_*, where *x_k_* and *y_k_* are nonzero moiety vectors. To decompose a composite moiety vector *l_k_*, we solved the mixed integer linear programming (MILP) problem

$$min\;1^{T}x_{k},$$ (14)

$$s.t.\;l_{k}=x_{k}+y_{k},$$ (15)

$$N^{T}x_{k}=0,$$ (16)

$$x_{k}\in {\mathbb{N}}_{0}^{m\times 1},$$ (17)

$$0<1^{T}x_{k}<1^{T}l_{k}.$$ (18)

We denote this problem by *P*_*k*_. The constraint in Eq 15 defines the solution vectors *x*_*k*_ and *y*_*k*_ as components of *l*_*k*_. The constraints in Eqs 16 and 17 correspond to Eqs 2 and 3 defining nonnegative integer conservation vectors (see Theoretical Framework, Section Moiety vectors). These constraints are implicit for *y*_*k*_ due to Eq 15. The constraint in Eq 18, when combined with Eq 15, ensures that *x*_*k*_ and *y*_*k*_ are both greater than zero. We chose to minimise the sum of elements in *x*_*k*_ but other objectives would also work. Problem *P*_*k*_ is infeasible for nondecomposable *l*_*k*_. We note that the solution vectors *x*_*k*_ and *y*_*k*_ might themselves be composite moiety vectors. To fully decompose the moiety matrix *L* we must therefore solve *P*_*k*_ iteratively until it is infeasible for all input moiety vectors. This process can be described with the algorithm,

1. Input $L\in {\mathbb{N}}_{0}^{m\times r}$. Initialise *L*′ = *L* and *D* = [ ], where [ ] denotes an empty matrix.

2. Set $r^{\prime }=\text{dim}\left(L_{1,:}^{\prime }\right)$ and L′′ = [ ], where $L_{1,:}^{\prime }$ denotes the first row of *L*′.

   If r′ ≥ 1, then go to Step 3,

   else, go to Step 5.

3. For *k* = 1: *r*′,

   denote *l*_*k*_ = *L*_:,*k*_,

   solve *P*_*k*_.

   If *P*_*k*_ is infeasible, set *D* = [*D*, *l*_*k*_],

   else, denote the solution of *P*_*k*_ by *x*_*k*_ and *y*_*k*_ and set *L*′′ = [*L*′′, *x*_*k*_, *y*_*k*_].

   Go to Step 4.

4. Set *L*′ = *L*′′ and go back to Step 2.

5. Output the fully decomposed moiety matrix $D\in {\mathbb{N}}_{0}^{m\times t}$.

The same algorithm can be used for nonnegative integer matrix factorisation of an elemental matrix and electron vector for a metabolic network.

### Implementation

We implemented the method presented here as an algorithmic pipeline in MATLAB (MathWorks, Natick, MA). This implementation is freely available as part of the COBRA toolbox [45] at https://github.com/opencobra/cobratoolbox (directory topology/conservedMoieties). Required inputs are an atom transition network and a stoichiometric matrix for a metabolic network. The method outputs moiety conservation relations both as moiety graphs and moiety vectors. All graphs are represented as incidence matrices. Support functions to generate atom transition networks (Section Generation of atom transition networks), classify moieties (Section Classification of moieties) and decompose moiety vectors (Section Decomposition of moiety vectors) are included with the core code. A tutorial on identification of conserved moieties in the dopamine synthesis network DAS is available at https://github.com/opencobra/cobratoolbox (directory topology/conservedMoieties/example), along with necessary data and MATLAB scripts that run through the example.

To compute the connected components of atom transition networks we used and implementation of Tarjan’s algorithm available as part of the Bioinformatics Toolbox for MATLAB (MathWorks, Natick, MA). This toolbox is not included with a standard installation of MATLAB. Users who do not have the Bioinformatics Toolbox can still run the pipeline with a free alternative to Tarjan’s algorithm to compute components of atom transition networks. If the Bioinformatics Toolbox is not installed in the MATLAB path, the pipeline calls a k-Nearest Neighbour algorithm in the MATLAB Network Routines toolbox by Bounova and Weck [46]. This toolbox is freely available with the COBRA toolbox. The k-Nearest Neighbour algorithm is considerably slower than Tarjan’s algorithm.

All code in the COBRA toolbox is distributed under a GNU General Public Licence and we encourage implementations of our method for other platforms than MATLAB. We have taken care to document and comment our code to facilitate such efforts.

## Supporting Information

S1 Appendix Mathematical definitions. Formal definitions of linear algebra and graph theory terms used or introduced in this work.
(PDF)

S1 Fig Conserved moieties and elementary metabolite units. Application of the algorithm presented in [34] to generate an elementary metabolite unit (EMU) reaction network from a moiety graph. (a) A toy metabolic network first presented in black [34] black. (b) An atom transition network for the toy metabolic network. (c) The moiety graph derived from the atom transition network. (d)-(f) Elementary metabolite unit reaction networks generated from the moiety graph. (d) Size 1 EMU species. (e) Size 2 EMU species. (f) Size 3 EMU species.
(PDF)
