# Supplementary material for: Identification of Conserved Moieties in Metabolic Networks by Graph Theoretical Analysis of Atom Transition Networks
Source: PLoS Comput Biol. 2016 Nov 21;12(11):e1004999. doi: 10.1371/journal.pcbi.1004999 (PMC5117560; doi:10.1371/journal.pcbi.1004999)
Supplement: S1 Appendix — Formal definitions of linear algebra and graph theory terms used or introduced in this work. (PDF) [file pcbi.1004999.s001.pdf]

Appendix: mathematical definitions

Linear algebra definitions

Definition 1. A *linear basis* for the left null space of a stoichiometric matrix $N \in \mathbb{R}^{m\times u}$ is given by a matrix $Z \in \mathbb{R}^{m\times r}$ with $N^\top Z = 0$ and $r = rank\,(Z) = m - rank\,(N)$.

Definition 2. A *convex basis* [1] for the left null space of a stoichiometric matrix $N \in \mathbb{R}^{m\times u}$ is given by a matrix $Z \in \mathbb{N}_0^{m\times h}$ with

(a) $N^\top Z = 0$,
(b) $h \geq rank\,(Z) = m - rank\,(N)$,
(c) $z_i \neq \alpha z_j + (1-\alpha)\, z_l \;\forall z_i, z_j, z_l \in Z \mid j \neq i, l \neq i; \alpha \in [0,1]$.

The columns of Z are called the *extreme rays* of the left null space.

Definition 3. A *nonnegative integer basis* for the left null space of a stoichiometric matrix $N \in \mathbb{R}^{m\times u}$ is given by a matrix $Z \in \mathbb{N}_0^{m\times r}$ with $N^\top Z = 0$ and $r = rank\,(Z) = m - rank\,(N)$.

Definition 4. A *nondecomposable nonnegative integer vector* is a vector $z_3 \in \mathbb{N}_0^m$ such that $z_3 \neq \alpha_1 z_1 + \alpha_2 z_2 \;\forall \alpha_1, \alpha_2 \in \mathbb{N}_+; z_1, z_2 \in \mathbb{N}_0^m; z_1, z_2 > 0$.

Graph theory definitions

Definition 5. [2] A *graph* $\Gamma = (\mathrm{X}(\Gamma), \mathrm{N}(\Gamma))$ is a mathematical structure consisting of a set of p nodes $\mathrm{X}(\Gamma) = \{\chi_1(\Gamma), \chi_2(\Gamma), \ldots, \chi_p(\Gamma)\}$ and a set of q edges $\mathrm{N}(\Gamma) = \{\nu_1(\Gamma), \nu_2(\Gamma), \ldots, \nu_q(\Gamma)\}$. Each edge $\nu_b(\Gamma) \in \mathrm{N}(\Gamma)$ connects two nodes $\{\chi_a(\Gamma), \chi_c(\Gamma)\} \in \mathrm{X}(\Gamma)$ which are called its endpoints.

Note 1. The endpoints of an edge are an *attribute* of that edge defined as $endpts\,(\nu_b(\Gamma)) = \{\chi_a(\Gamma), \chi_c(\Gamma)\}$. The graph concept can be extended by assigning additional attributes to edges and nodes.

Definition 6. [2] A *directed graph* $\mathrm{A} = (\mathrm{X}(\mathrm{A}), \mathrm{N}(\mathrm{A}))$ is a graph where each edge $\nu_b(\mathrm{A}) \in \mathrm{N}(\mathrm{A})$ is directed from a tail node $\chi_a(\mathrm{A}) = tail\,(\nu_b(\mathrm{A}))$ to a head node $\chi_c(\mathrm{A}) = tail\,(\nu_b(\mathrm{A}))$ where $\{\chi_a(\mathrm{A}), \chi_c(\mathrm{A})\} = endpts\,(\nu_b(\mathrm{A}))$.

Note 2. The *underlying graph* of a directed graph is obtained by removing all assignments of edge directions.

Definition 7. [3] A *directed hypergraph* $\mathrm{M} = (\mathrm{X}(\mathrm{M}), \mathrm{N}(\mathrm{M}))$ consists of a set of m nodes $\mathrm{X}(\mathrm{M}) = \{\chi_1(\mathrm{M}), \chi_2(\mathrm{M}), \ldots, \chi_m(\mathrm{M})\}$ and a set of n hyperedges $\mathrm{N}(\mathrm{M}) = \{\nu_1(\mathrm{M}), \nu_2(\mathrm{M}), \ldots, \nu_n(\mathrm{M})\}$. Each hyperedge $\nu_j(\mathrm{M}) \in \mathrm{N}(\mathrm{M})$ can connect an arbitrary number of nodes, i.e., $endpts\,(\nu_j(\mathrm{M})) = \bar{\bar{\chi}}(\mathrm{M})$ where $\bar{\bar{\chi}}(\mathrm{M}) \subseteq \mathrm{X}(\mathrm{M})$ and $\mid \bar{\bar{\chi}}(\mathrm{M}) \mid \geq 1$. Each hyperedge is directed from a set of tail nodes $tails\,(\nu_j(\mathrm{M})) \subseteq \bar{\bar{\chi}}(\mathrm{M})$ to a set of head nodes $heads\,(\nu_j(\mathrm{M})) \subseteq \bar{\bar{\chi}}(\mathrm{M})$.

Definition 8. A *metabolic network* is a directed hypergraph $\mathrm{M} = (\mathrm{X}(\mathrm{M}), \mathrm{N}(\mathrm{M}))$ where each node $\chi_i(\mathrm{M}) \in \mathrm{X}(\mathrm{M})$ represents a metabolite and each hyperedge $\nu_j(\mathrm{M}) \in \mathrm{N}(\mathrm{M})$ represents a reaction. Each reaction $\nu_j(\mathrm{M})$ is directed from a set of substrates $\chi_a(\mathrm{A}) \in tails\,(\nu_b(\mathrm{A}))$, to a set of products $\chi_c(\mathrm{A}) \in heads\,(\nu_b(\mathrm{A}))$.

Definition 9. An *atom transition network* is a directed graph $\mathrm{A} = (\mathrm{X}(\mathrm{A}), \mathrm{N}(\mathrm{A}))$ where each node $\chi_a(\mathrm{A}) \in \mathrm{X}(\mathrm{A})$ represents an atom in a particular metabolite and each edge $\nu_b(\mathrm{A}) \in \mathrm{N}(\mathrm{A})$ represents an atom transition in a particular metabolic reaction. Each atom transition $\nu_b(\mathrm{A})$ is directed from a substrate atom $\chi_a(\mathrm{A}) = tail\,(\nu_b(\mathrm{A}))$, to a product atom $\chi_c(\mathrm{A}) = head\,(\nu_b(\mathrm{A}))$. Each atom $\chi_a(\mathrm{A})$ is associated with its parent metabolite $\chi_i(\mathrm{M})$ in the metabolic network M through its metabolite attribute $met\,(\chi_a(\mathrm{A})) = \chi_i(\mathrm{M})$. Each atom transition is likewise associated with its parent reaction $\nu_j(\mathrm{M})$ in M through its reaction attribute $rxn\,(\nu_b(\mathrm{A})) = \nu_j(\mathrm{M})$.

Definition 10. [2] A *self-loop* is an edge $\nu_b(\Gamma)$ that connects a node $\chi_a(\Gamma)$ to itself such that $endpts\,(\nu_b(\Gamma)) = \chi_a(\Gamma)$

Definition 11. [2] A *multi-edge* $\bar{\bar{\nu}}(\Gamma) \subseteq \mathrm{N}(\Gamma)$ is a set of two or more edges connecting the same two nodes $\chi_a(\Gamma)$ and $\chi_c(\Gamma)$ such that $endpts\,(\nu_b(\Gamma)) = \{\chi_a(\Gamma), \chi_c(\Gamma)\}\, \forall \nu_b(\Gamma) \in \bar{\bar{\nu}}(\Gamma)$.

Note 3. Definitions 5 and 6 do not preclude self-loops or multi-edges.

Definition 12. [2] A *simple graph* has no self-loops or multi-edges.

Note 4. The *underlying simple graph* of a general graph (Definition 5) is obtained by removing any self-loops and merging any multi-edges into single edges.

Definition 13. [2]A graph $\Gamma = (\mathrm{X}(\Gamma), \mathrm{N}(\Gamma))$ is *connected* if every node $\chi_a(\Gamma) \in \mathrm{X}(\Gamma)$ is reachable from every other node $\chi_c(\Gamma) \in \mathrm{X}(\Gamma)$.

Definition 14. [2]A directed graph is *connected* if the underlying graph is connected.

Definition 15. [2] A *connected component* of a directed graph A is a maximal connected subgraph $\kappa = (\mathrm{X}(\kappa), \mathrm{N}(\kappa))$ where $\mathrm{X}(\kappa) \subseteq \mathrm{X}(\mathrm{A})$ and $\mathrm{N}(\kappa) \subseteq \mathrm{N}(\mathrm{A})$.

Definition 16. [2] An *isomorphism* between two graphs $\Gamma_1 = (\mathrm{X}(\Gamma_1), \mathrm{N}(\Gamma_1))$ and $\Gamma_2 = (\mathrm{X}(\Gamma_2), \mathrm{N}(\Gamma_2))$ is a vertex bijection $f\colon \mathrm{X}(\Gamma_1) \to \mathrm{X}(\Gamma_2)$ that is structure-preserving.

Note 5. A vertex bijection f, between two simple graphs Γ_1 and Γ_2, is structure-preserving if it preserves the adjacency and nonadjacency of all nodes, i.e., if any two nodes $\chi_a(\Gamma_2) = f(\chi_a(\Gamma_1))$ and $\chi_c(\Gamma_2) = f(\chi_c(\Gamma_1))$ in $\mathrm{X}(\Gamma_2)$ are adjacent in Γ_2 if and only if $\chi_a(\Gamma_1)$ and $\chi_c(\Gamma_1)$ in $\mathrm{X}(\Gamma_1)$ are adjacent in Γ_1. The concept of a structure-preserving vertex bijection can be extended to other types of graphs by specifying additional attributes that should be preserved, e.g., an isomorphism between two directed graphs A_1 and A_2, with no self-loops or multi-edges, is a vertex bijection $f\colon \mathrm{X}(\mathrm{A}_1) \to \mathrm{X}(\mathrm{A}_2)$ that preserves the adjacency and nonadjacency of all nodes as well as the directions of all edges.

Definition 17. A vertex bijection $f\colon \mathrm{X}(\kappa_1) \to \mathrm{X}(\kappa_2)$ between two components κ_1 and κ_2 of an atom transition network A is *structure preserving* if and only if it preserves the metabolite attribute of every node, i.e., $met(\chi_a(\mathrm{A})) = met(\chi_c(\mathrm{A}))$ $\forall \chi_a(\mathrm{A}) \in \mathrm{X}(\kappa_1), \chi_c(\mathrm{A}) \in \mathrm{X}(\kappa_2) \mid \chi_c(\mathrm{A}) = f(\chi_a(\mathrm{A}))$.

Note 6. The nature of chemical reactions ensures that a vertex bijection that preserves the metabolite attribute of every node also preserves all other structural elements including the adjacency and nonadjacency of every node, and the direction and reaction attribute of every edge.

Definition 18. Two atoms $\chi_a(\mathrm{A}) \in \mathrm{X}(\kappa_1)$ and $\chi_c(\mathrm{A}) \in \mathrm{X}(\kappa_2)$ in two distinct components κ_1 and κ_2 of an atom transition network A are *equivalent* if and only if $\chi_c(\mathrm{A}) = f(\chi_a(\mathrm{A}))$ where $f\colon \mathrm{X}(\kappa_1) \to \mathrm{X}(\kappa_2)$ satisfies definition 17.

Definition 19. A *conserved moiety* is a maximal set of equivalent atoms in an atom transition network.

Definition 20. A *moiety graph* is a directed graph $\lambda = (\mathrm{X}(\lambda), \mathrm{N}(\lambda))$ representing a moiety conservation relation for a metabolic network M. Each node $\chi_a(\lambda) \in \mathrm{X}(\lambda)$ represents an instance of a conserved moiety in a particular metabolite $\chi_i(\mathrm{M}) = met(\chi_a(\lambda))$. Each edge $\nu_b(\lambda) \in \mathrm{N}(\lambda)$, with $\chi_a(\lambda) = tail(\nu_b(\lambda))$ and $\chi_c(\lambda) = head(\nu_b(\lambda))$, represents conservation of a moiety between a substrate $met(\chi_a(\lambda))$ and a product $met(\chi_c(\lambda))$. Each node is associated with a set of atoms in the atom transition network A through its atom attribute $atoms(\chi_a(\lambda)) = \bar{\bar{\chi}}_a(\mathrm{A})$ where $\bar{\bar{\chi}}_a(\mathrm{A}) \subseteq \mathrm{X}(\mathrm{A})$ and $\chi_c(\mathrm{A}) = f(\chi_a(\mathrm{A}))\, \forall \chi_a(\mathrm{A}), \chi_c(\mathrm{A}) \in \bar{\bar{\chi}}_a(\mathrm{A})$. Each edge is associated with a set of atom transitions in A through its transition attribute $transitions(\nu_b(\lambda)) = \bar{\bar{\nu}}_b(\mathrm{A})$ where $\bar{\bar{\nu}}_b(\mathrm{A}) \subseteq \mathrm{N}(\mathrm{A})$ and $tail(\nu_b(\mathrm{A})) \in tail(\nu_b(\lambda)), head(\nu_b(\mathrm{A})) \in head(\nu_b(\lambda))\, \forall \nu_b(\mathrm{A}) \in \bar{\bar{\nu}}_b(\mathrm{A})$.

Note 7. A moiety graph λ is obtained from an atom transition network A by merging all isomorphic components into a single graph.

Definition 21. A *moiety vector* is a nonnegative integer vector $l_1 \in \mathbb{N}_0^m$ representing a moiety conservation relation for a metabolic network M with internal stoichiometric matrix $N \in \mathbb{Z}^{m \times u}$. It satisfies,

(a) $N^\top l_1 = 0$,
(b) $l_{1,i} = |\chi_a(\lambda) \in \mathrm{X}(\lambda) \mid met(\chi_a(\lambda)) = \chi_i(\mathrm{M})|$, where λ is a moiety graph.

A moiety vector is *composite* if it also satisfies,

(c) $l_1 = \alpha_2 l_2 + \alpha_3 l_3$ where $\alpha_2, \alpha_3 \in \mathbb{N}_+$ and l_2, l_3 satisfy the definition of a moiety vector above.

A moiety vector is *nondecomposable* if it is not composite.

Note 8. A moiety vector l is derived from a moiety graph λ by setting element i of l to the number of nodes $\chi_a(\lambda)$ in λ where $met(\chi_a(\lambda)) = \chi_i(\mathrm{M})$. The elements of l therefore correspond to the incidence of a conserved moiety in each metabolite of the metabolic network M. A nondecomposable moiety vector cannot be decomposed into smaller moiety vectors whereas a composite moiety vector can.

Definition 22. A *moiety basis* for the left null space of a stoichiometric matrix $N \in \mathbb{R}^{m \times u}$ is given by a matrix $L \in \mathbb{N}_0^{m \times r}$ with $N^\top L = 0$, $r = rank(L) = m - rank(N)$, and $L_{:,k}$ satisfying definition 21 for all $k \in [1, r]$.

Definition 23. [2] The *incidence matrix of a graph* $\Gamma = (\mathrm{X}(\Gamma), \mathrm{N}(\Gamma))$ with p nodes and q edges is a matrix $G \in \{0,1,2\}^{p \times q}$ where

$$G_{ab} = \begin{cases} 0 & \Leftrightarrow \chi_a \notin endpts(\nu_b), \\ 1 & \Leftrightarrow \chi_a \in endpts(\nu_b), \\ 2 & \Leftrightarrow \chi_a = endpts(\nu_b). \end{cases} \tag{1}$$

Definition 24. [2] The *incidence matrix of a directed graph* $\mathrm{A} = (\mathrm{X}(\mathrm{A}), \mathrm{N}(\mathrm{A}))$ with p nodes and q directed edges is a matrix $A \in \{-1, 0, 1, 2\}^{p \times q}$ where

$$A_{ab} = \begin{cases} 0 & \Leftrightarrow \chi_a \notin \mathit{endpts}(\nu_b), \\ -1 & \Leftrightarrow \chi_a = \mathit{tail}(\nu_b), \\ 1 & \Leftrightarrow \chi_a = \mathit{head}(\nu_b), \\ 2 & \Leftrightarrow \chi_a = \mathit{endpts}(\nu_b). \end{cases} \tag{2}$$

Definition 25. The *incidence matrix of a directed hypergraph* $\mathrm{M} = (\mathrm{X}(\mathrm{M}), \mathrm{N}(\mathrm{M}))$ with m nodes and n hyperedges is a matrix $M \in \mathbb{Z}^{m \times n}$ where

$$M_{ij} = \begin{cases} 0 & \Leftrightarrow \chi_i \notin \mathit{endpts}(\nu_j), \\ < 0 & \Leftrightarrow \chi_i \in \mathit{tails}(\nu_j), \\ > 0 & \Leftrightarrow \chi_i \in \mathit{heads}(\nu_j). \end{cases} \tag{3}$$

References

[1] Famili I, Palsson BØ. The convex basis of the left null space of the stoichiometric matrix leads to the definition of metabolically meaningful pools. Biophys J. 2003; 85: 16–26.

[2] Gross JL, Yellen J. Graph Theory and Its Applications. 2nd ed. Boca Raton: Chapman and Hall/CRC; 2005.

[3] Klamt S, Haus UU, Theis F. Hypergraphs and cellular networks. PLoS Computational Biology. 2009; 5: e1000385.
