# Supplementary material for: Identification of Conserved Moieties in Metabolic Networks by Graph Theoretical Analysis of Atom Transition Networks
Source: PLoS Comput Biol. 2016 Nov 21;12(11):e1004999. doi: 10.1371/journal.pcbi.1004999 (PMC5117560; doi:10.1371/journal.pcbi.1004999)
Supplement: S1 Fig — Application of the algorithm presented in [34] to generate an elementary metabolite unit (EMU) reaction network from a moiety graph. (a) A toy metabolic network first presented in black [34] black. (b) An atom transition network for the toy metabolic network. (c) The moiety graph derived from the atom transition network. (d)-(f) Elementary metabolite unit reaction networks generated from the moiety graph. (d) Size 1 EMU species. (e) Size 2 EMU species. (f) Size 3 EMU species. (PDF) [file pcbi.1004999.s002.pdf]

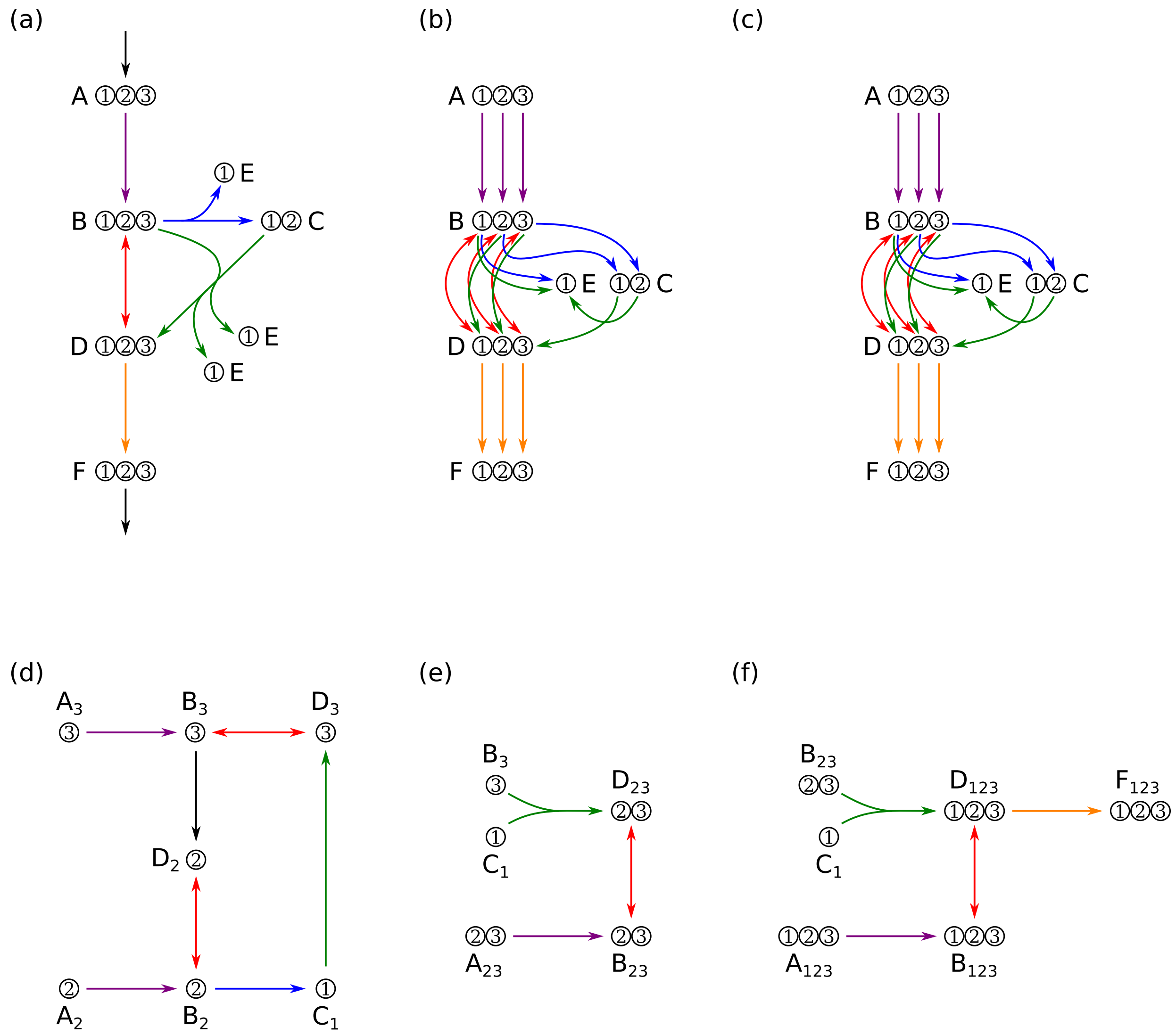

Figure 1: **Conserved moieties and elementary metabolite units.** (a) A toy metabolic network presented in [1]. The network includes six metabolites and five internal reactions. (b) An atom transition network for the metabolic network in (a). The atom transition network was generated based on atom mappings provided in [1]. Each edge can be matched to its corresponding reaction in (a) by its edge colour. (c) The moiety graph derived from the atom transition network in (b). Internal reactions of the metabolic network only conserve one moiety. Each instance of the moiety consists of a single atom. The moiety graph is therefore identical to the atom transition network for this toy metabolic network. (d)-(f) Elementary metabolite unit reaction networks for EMU species of increasing sizes. The networks were generated by applying the algorithm presented in [1] to the moiety graph in (c). The three networks include a total of 14 EMU species (d) Size 1. (e) Size 2. (f) Size 3. Exactly the same EMU networks were obtained from the atom transition network in (b).

References

[1] Antoniewicz MR, Kelleher JK, Stephanopoulos G. Elementary metabolite units (EMU): a novel framework for modeling isotopic distributions. Metab Eng. 2007; 9: 68–86.
